# Insuficiência Tricúspide e Mortalidade em Pacientes Submetidos à Troca da Valva Aórtica Transcateter: Uma Revisão Sistemática e Metanálise

**DOI:** 10.36660/abc.20220319

**Published:** 2023-07-27

**Authors:** Bruna Olandoski Erbano, Nicolle Amboni Schio, Renato Delascio Lopes, Tiago Costa Bignoto, Marcia Olandoski, Raquel Silva Brito da Luz, Guilherme Dagostin de Carvalho, Lucas Henrique Olandoski Erbano, Auristela Isabel de Oliveira Ramos, Fausto Feres, José Rocha Faria, Cristina Pellegrino Baena, Dimytri Alexandre de Alvim Siqueira

**Affiliations:** 1 Programa de Pós-Graduação Instituto Dante Pazzanese de Cardiologia Universidade de São Paulo São Paulo SP Brasil Programa de Pós-Graduação do Instituto Dante Pazzanese de Cardiologia associado à Universidade de São Paulo, São Paulo, SP – Brasil; 2 Pontifícia Universidade Católica do Paraná Curitiba PR Brasil Pontifícia Universidade Católica do Paraná, Curitiba, PR – Brasil; 3 Duke University Hospital Durham North Carolina EUA Duke University Hospital, Durham, North Carolina – EUA; 4 Instituto Dante Pazzanese de Cardiologia São Paulo SP Brasil Instituto Dante Pazzanese de Cardiologia, São Paulo, SP – Brasil

**Keywords:** Substituição da Valva Aórtica Transcateter, Insuficiência da Valva Tricúspide, Mortalidade, Estenose da Valva Aórtica

## Abstract

**Fundamento:**

A extensão do dano cardíaco associada à estenose aórtica tem importantes implicações prognósticas após a substituição da valva aórtica transcateter (TAVR). Contudo, ainda não está claro qual é o papel da insuficiência tricúspide (IT) nesse cenário clínico.

**Objetivos:**

Explorar a associação entre IT e mortalidade em pacientes submetidos a TAVR e avaliar as alterações na gravidade da IT após a TAVR e sua relação com mortalidade de curto e médio prazo.

**Métodos:**

Foram feitas pesquisas em bases de dados relevantes de artigos publicados do início até agosto de 2020. Dos 414 estudos triados, selecionamos 24 que relataram o grau de IT pré- ou pós-TAVR. O desfecho primário foi mortalidade por todas as causas, e foram conduzidos modelos de metanálise de efeitos aleatórios (a um nível de significância de 5%).

**Resultados:**

Dezessete estudos relataram associações entre IT pré-TAVR e mortalidade por todas as causas (> 45.000 participantes), e 13 avaliaram a gravidade da IT pós-TAVR (709 participantes). A IT basal moderada/grave foi associada a maior mortalidade por todas as causas em 30 dias [razão de risco (RR) 1,65; intervalo de confiança (IC) 95% 1,20-2,29] e 1,2 ano (RR 1,56; IC95% 1,31-1,84). Após a TAVR, 43% dos pacientes apresentaram redução de pelo menos um grau na IT (30 dias, IC95% 30-56%), que se sustentou em 12,5 meses em 44% dos participantes (IC95% 35-52%).A persistência de IT significativa foi associada a um aumento de duas vezes na mortalidade por todas as causas (RR 2,12; IC95% 1,53-2,92).

**Conclusões:**

A IT significativa pré-TAVR está associada a maior mortalidade. Ainda que a gravidade da IT possa melhorar, a persistência de IT significativa após a TAVR está fortemente associada ao aumento da mortalidade. Nossos achados destacam a importância de uma avaliação detalhada da IT pré- e pós-TAVR e podem ajudar a identificar pacientes que possam se beneficiar de uma vigilância mais cuidadosa nesse cenário.

**Figura Central:**
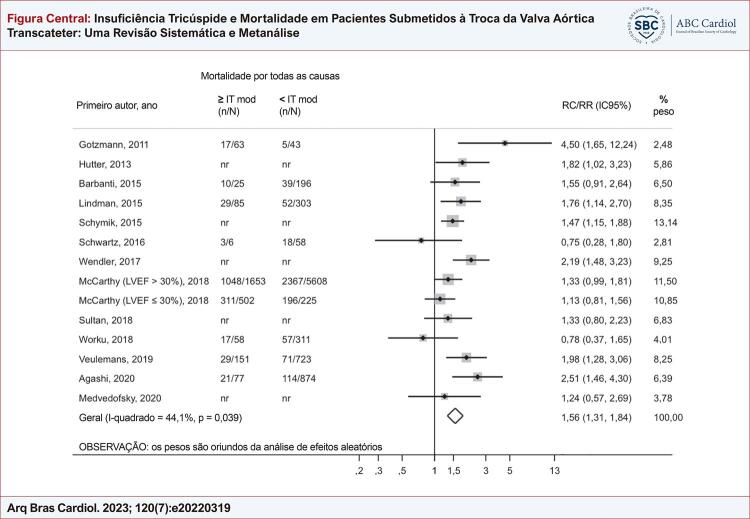
: Insuficiência Tricúspide e Mortalidade em Pacientes Submetidos à Troca da Valva Aórtica Transcateter: Uma Revisão Sistemática e Metanálise

## Introdução

Nas últimas duas décadas, a mortalidade após a substituição da valva aórtica transcateter (TAVR) diminuiu.^1^ Porém, nos 5 anos após a TAVR, taxa de mortalidade de quase 50% é atribuída a causas cardiovasculares.^2^ Conforme descrito por Généreux et al.,^3^ a extensão do dano cardíaco secundário à estenose aórtica (EA) tem importantes implicações prognósticas após a substituição da valva aórtica (AVR). Em relação a isso, tem-se estudado extensivamente a associação da gravidade da insuficiência mitral (IM) e de maiores taxas de mortalidade após a TAVR.^4^ Contudo, em um subgrupo de IM leve a moderada, a insuficiência tricúspide (IT) foi o fator proeminente associado a um pior prognóstico.^5^

Na verdade, a EA em conjunto com a IT moderada/grave e/ou hipertensão pulmonar está associada a 21,3% da mortalidade por todas as causas em 1 ano, independentemente do tratamento da EA.^3^ Contudo, um grande registro chegou à conclusão que a IT só foi preditiva de morte após a TAVR em pacientes com mais de 30% de fração de ejeção do ventrículo esquerdo (FEVE),^6^ o que significa que a interação entre essas valvopatias permanece incerta. Porém, sabe-se pouco sobre as alterações na gravidade da IT ao longo do tempo após a TAVR. Os objetivos desta revisão sistemática e metanálise foram explorar a associação entre a IT e a mortalidade em pacientes submetidos a TAVR e avaliar as alterações na gravidade da IT após a TAVR e sua relação com mortalidade em curto e médio prazo.

## Métodos

### Estratégia de pesquisa

**Figura 1:**
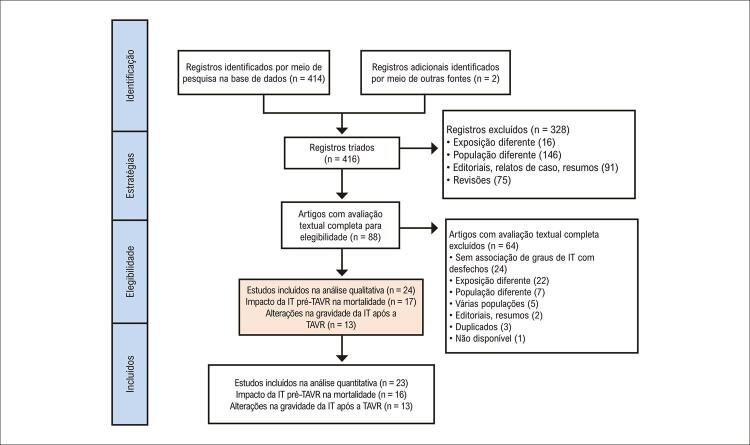
– Fluxograma do PRISMA. PRISMA: Preferred Reporting Items for Systematic Reviews and Meta-Analyses (Itens de relatório preferidos para revisões sistemáticas e metanálises). IT: insuficiência tricúspide; TAVR: substituição da valva aórtica transcateter.

Este estudo foi conduzido de acordo com as recomendações do PRISMA ( *Preferred Reporting Items for Systematic Reviews and Meta-Analyses* /Itens de relatório preferidos para revisões sistemáticas e metanálises),^7^ do MOOSE ( *Meta-analyses of Observational Studies in Epidemiology* /Metanálises de estudos observacionais em epidemiologia)^8^ e da Cochrane^9^ e foi considerado isento de aprovação por um conselho de revisão institucional. Artigos relevantes foram pesquisados em cinco bases de dados eletrônicas (MEDLINE/PubMed, SCOPUS, EMBASE, Web of Science e LILACS) usando os seguintes termos em inglês: *TAVR OR AND tricuspid regurgitation AND prognosis/mortality* (TAVR OU E IT E prognóstico/mortalidade) ( Material complementar 1 ). A busca foi realizada do início até agosto de 2020, sem restrições de idioma. A Figura 1 exibe o diagrama de fluxo do PRISMA. Dois pares de autores examinaram independentemente todos os títulos e resumos, e os registros relevantes foram selecionados para uma análise completa. As divergências foram resolvidas por consenso após consulta a um revisor sênior. As listas de referências dos artigos obtidos e revisões relevantes também foram triadas. A estatística Kappa foi usada para determinar o grau de concordância entre os revisores.

### Critérios de elegibilidade

Incluímos estudos que: (1) avaliaram pacientes com TAVR devido a EA; (2) relataram graus de IT por ecocardiografia (pré- ou pós-TAVR); (3) relataram mortalidade por todas as causas como desfecho primário, além de mortalidade cardiovascular e hospitalização por insuficiência cardíaca (ICa) como desfechos secundários, de acordo com o grau de IT. Excluímos estudos que: (1) incluíram exclusivamente pacientes com valva aórtica bicúspide e EA ou aqueles submetidos a procedimentos de valve-in-valve; (2) não avaliaram os graus de IT conforme recomendado pelas diretrizes de ecocardiografia;^10^ ou (3) apresentaram relatórios pouco claros sobre variáveis, desfechos de interesse ou desfechos combinados, impossibilitando a análise dos dados. Para a análise quantitativa, excluímos estudos que avaliaram exclusivamente populações de subgrupos que eram diferentes dos participantes da revisão. Em casos em que a mesma população de pacientes foi relatada em várias publicações, selecionamos o estudo com a maior amostra. Foram excluídos relatos de casos, resumos, revisões, editoriais e relatórios de conferências.

### Extração de dados

Os dados foram coletados por três autores usando uma planilha de extração de dados predefinida ( Material complementar 2 ), a qual incluía detalhes do estudo, dados demográficos basais do paciente, características clínicas e ecocardiográficas e desfechos de interesse. As divergências foram resolvidas por consenso após consulta a um autor sênior. Caso as características basais do paciente estivessem separadas por grupos, sempre que possível, agrupamos os dados atribuíveis a toda a população usando a média e desvio padrão (DP).^11^

### Mortalidade e gravidade da IT

O desfecho primário foi definido como a incidência de mortalidade por todas as causas de acordo com os graus basais de IT. Os desfechos secundários incluíram mortalidade cardíaca e hospitalização por ICa. Os estudos foram separados entre seguimento de curto prazo (desfechos avaliados até a alta ou 30 dias após a TAVR) e seguimento de médio prazo (desfechos avaliados mais de 30 dias após a TAVR).

Os graus de IT avaliados por ecocardiografia foram classificados como ausente/mínimo, leve, moderada ou grave. Nossas análises primárias compararam graus de IT moderada/grave com graus de IT ausente/mínimo/leve. A associação de graus incrementais de IT e sobrevida também foi examinada por meio da comparação do risco de mortalidade por IT ausente/mínimo com IT leve, moderada e grave (análise secundária). Um estudo^12^ comparou a IT grave com não grave, o que foi incluído na análise primária.

Para nossas análises adicionais, as melhoras no grau de IT foram definidas por alterações de pelo menos um grau desde a linha de base até o pós-TAVR. Metanálises também foram feitas separadamente por tempos de seguimento, e a mortalidade por todas as causas foi comparada entre pacientes cuja gravidade da IT melhorou após a TAVR e aqueles em que a IT piorou ou permaneceu inalterada.

### Avaliações de qualidade e de risco de viés

A escala de Newcastle-Ottawa^13^ foi usada para avaliar o risco de viés. Dois revisores independentes classificaram os estudos como apresentando risco de viés baixo (nove estrelas), médio (sete ou oito estrelas) ou alto (seis ou menos estrelas). Todas as divergências foram resolvidas por consenso.

### Análise estatística

Como apenas estudos observacionais foram incluídos, as estimativas agrupadas de mortalidade por todas as causas e intervalos de confiança (IC) de 95% dos estudos incluídos foram obtidas por metanálises de efeitos aleatórios (método DerSimonian e Laird, com as estimativas de heterogeneidade obtidas do método Mantel-Haenszel).^9^ Foram extraídos de cada estudo, a razão de chances (RC) mais amplamente ajustada ou (quando não disponível) não ajustada, a razão de risco (RR) e os IC95% associados. Caso as estimativas de risco não estivessem disponíveis, obtivemos os dados relevantes: entrando em contato com os autores; fazendo o cálculo manualmente com base nas informações disponíveis; ou calculando a RR não ajustada com base nas curvas de Kaplan-Meier publicadas.^14^ Presumimos que a RR e a RC se aproximavam da mesma medida de risco.^9^ Em um caso,^6^ a RR correspondente a IT ausente/leve vs. moderada/grave foi calculada com base na RR de outras comparações (IT ausente/mínimo vs. leve, moderada e grave). As estimativas agrupadas das diferenças médias nas proporções pré- e pós-TAVR dos graus de IT moderada/grave foram calculadas para avaliar as mudanças na IT desde a linha de base até o seguimento. A heterogeneidade entre os estudos foi avaliada com a estatística I^2^ e classificada como: < 25% indicava baixa heterogeneidade, e > 75% indicava alta heterogeneidade. As análises de sensibilidade foram realizadas por meio da análise *leave-one-out* , separando estimativas de risco ajustadas e não ajustadas. Análises de metarregressão foram usadas para testar covariáveis importantes para a influência de modificadores de efeito potencial. O viés de publicação foi avaliado pela simetria do gráfico de funil e pelo teste de Egger^15^ (o p-valor > 0,05 indicou que não havia viés significativo). Todas as análises foram realizadas usando o software estatístico Stata versão 14.1 (StataCorp LP, College Station, Texas, EUA).

## Resultados

### Seleção de estudo

As buscas eletrônicas resultaram em 414 estudos não duplicados; e dois estudos adicionais foram selecionados manualmente. Após a avaliação do título e do resumo, 88 estudos foram selecionados para avaliação do texto completo [Kappa = 0,86 (IC95% 0,79-0,92)]. Por fim, 24 relatórios foram considerados elegíveis e foram incluídos em nossa revisão sistemática: 17 avaliando o impacto da IT basal na mortalidade por todas as causas após a TAVR^5 , 6 , 12 , 16 - 29^ e 13 na análise adicional.^17 , 20 , 22 - 24 , 29 - 36^ Para as análises quantitativas, excluímos um artigo,^5^ que avaliava um subgrupo específico de pacientes com IM leve a moderada, porque ele considerava uma população divergente com uma proporção provavelmente maior de pacientes com IT primária. Um resumo dos 17 estudos selecionados é apresentado na Tabela 1 . Oito estudos relataram dados sobre desfechos de 30 dias, e 14 estudos relataram dados sobre seguimento de médio prazo (média de 1,2 ano).

**Tabela 1:** – Principais características dos estudos incluídos

Primeiro autor, ano	Região	Número de	Desenho	Período	N°. de participantes	Tipo de	Acesso			Mortalidade
(N°. de ref.)		centros	do estudo	de inclusão	submetidos a TAVR	valva (%)	transfemoral (%)	Gravidade da IT (%)	Seguimento	por todas as causas (%)*
Agasthi, 2020	EUA	3	Retrospectivo	Janeiro de 2012 a	954	BEV 745 (78)	726 (76)	< mod = 877 (92)	1 ano	135 (14)
(16)	(Hospitais Mayo Clinic)			Junho de 2017		SEV 209 (22)		≥ mod = 77 (8)		
Amat-santos, 2018	Espanha	6	Retrospectivo	Agosto de 2007 a	813	BEV 194 (24)	813 (100)	< 2 = 602 (74)	6 meses	84 (10)
(5)				Janeiro de 2015		SEV 608 (76)		≥ 2 = 208 (26)		
Barbanti, 2015	Canadá	1	Retrospectivo	Janeiro de 2007 a	518	BEV 483 (93)	343 (66)	< mod = 439 (85)	30 dias e	118 (23)
(17)				Agosto de 2012		SEV 35 (7)		≥ mod = 79 (15)	2 anos	
Barvalia, 2017	EUA	1	Retrospectivo	2012 a 2015	460	BEV 280 (61)	330 (72)	Leve = 352 (76)	30 dias	25 (5)
(18)	(New Jersey)					SEV 180 (39)		Moderada = 32 (7)		
								Grave = 43 (9)		
Gotzmann, 2011	Alemanha	1	Prospectivo	Junho de 2008 a	145	SEV 145 (100)	140 (96)	Leve = 43 (30)	6 meses	23 (16)
(19)	(Bochum)			Setembro de 2010				Moderada = 46 (32)		
								Grave = 17 (12)		
Hutter, 2013	Alemanha	1	Prospectivo	Junho de 2007 a	268	BEV 74 (28)	194 (72)	< mod = 197 (78)	30 dias e	108 (40)†
(20)	(Munique)			Agosto de 2009		SEV 194 (72)		≥ mod = 54 (21)	2 anos	
Kjonas, 2019	Noruega	2	Prospectivo	Fevereiro de 2010 a	218	BEV 170 (78)	122 (56)	< mod = 168 (77)	30 dias	19 (9)
(21)				Junho de 2013		SEV 48 (22)		≥ mod = 45 (21)		
Lindman, 2015	EUA e	57	Prospectivo	Dezembro de 2011 a	507	BEV 507 (100)	507 (100)	Leve = 372 (73)	1 ano	112 (22)
(22)	Canadá			Novembro de 2013				Moderada = 117 (23)		
								Grave = 18 (3)		
McCarthy, 2018	EUA	365	Retrospectivo	Novembro de 2011 a	34.576	nr	nr	Ausente/mínimo = 6.772 (19)	Intra-hospitalar	3.993 (11)
(6)	(Registro do STS)			Março de 2015				Leve = 19.393 (56)	e 1 ano	
								Moderada = 6.687 (19)		
								Grave = 1.724 (5)		
Medvedofsky, 2020	EUA	1	Retrospectivo	Maio de 2007 a	334	nr	nr	Não grave = 329 (98)	1 ano	80 (24)
(12)	(Washington)			Março de 2014				Grave = 5 (2)		
Omar, 2020	EUA	1	Retrospectivo	Agosto de 2014 a	174	BEV 76 (44)	166 (95)	Leve = 124 (71)	Intra-hospitalar	13 (7)
(23)	(Flórida)			Janeiro de 2017		SEV 98 (56)		Moderada = 34 (19)		
								Grave = 16 (9)		
Schwartz, 2016	Israel	1	Retrospectivo	Março de 2009 a	519	nr	nr	Leve = 460 (89)	30 dias e	108 (21)
(24)				Junho de 2014				Moderada = 44 (8)	1,5 ± 1,17 anos	
								Grave = 15 (3)		
Schymik, 2015	Multicêntrico	99	Prospectivo	Julho de 2010 a	2.688	BEV 2.688 (100)	1.685 (62)	< mod = 2.089 (85)	1 ano	515 (19)
(25)	(17 países)			Novembro de 2011				≥ mod = 343 (14)		
Sultan, 2018	EUA	1	Retrospectivo	Julho de 2011 a	457	BEV 369 (80)	337 (74)	< mod = 387 (85)	23 ± 14 meses	103 (22)
(26)	(Pittsburgh)			Janeiro de 2016		SEV 87 (20)		≥ mod = 70 (15)		
Veulemans, 2019	Alemanha	1	Retrospectivo	2009 a 2018	874	nr	737 (84)	< mod = 723 (83)	1 ano	100 (11)
(27)	(Düsseldorf)							≥ mod = 151 (17)		
Wendler, 2017	Europa	80	Prospectivo	Julho de 2014 a	1.946	BEV 1.946 (100)	1.694 (87)	< mod = 1.470 (89)	1 ano	245 (13)
(28)				Outubro de 2015				≥ mod = 180 (11)		
Worku, 2018	EUA	1	Prospectivo	2009 a 2014	369	BEV 359 (97)	230 (62)	Leve = 311 (84)	30 dias e	74 (20)
(29)	(New York)					SEV 10 (3)		Moderada = 28 (7)	610 dias (média)	
								Grave = 30 (8)		

Os valores são média ± DP ou n (%). Todos os estudos consideraram valores < 0,05 para indicar a significância estatística. BEV: valva aórtica transcateter expansível por balão; mod: insuficiência tricúspide moderada; N°.: número; nr: não relatado; ref.: referência; IT: insuficiência tricúspide; SEV: valva aórtica transcateter autoexpansível; TAVR: substituição da valva aórtica transcateter. * Taxa de mortalidade por todas as causas relatada no seguimento mais longo. † Mortalidade por todas as causas em 1 ano de seguimento.

### População do estudo

Foram incluídos mais de 45.000 pacientes de aproximadamente 600 centros de saúde em todo o mundo. A idade média foi de 81,7 ± 8,5 anos, com 52% da população sendo do sexo feminino, e a pontuação média da Society of Thoracic Surgeons (STS) foi de 8,2 ± 6,0. Aproximadamente 22% dos pacientes apresentavam IT moderada ou grave na linha de base. As características clínicas e os parâmetros ecocardiográficos basais estão listados nas Tabelas 2 e 3 .

**Tabela 2:** – Características clínicas dos pacientes incluídos

Primeiro autor, ano	N°. de participantes										Classe funcional do
(N°. de ref.)	submetidos a TAVR	Idade (anos)	Feminino (%)	Escore STS†	HT (%)	DM (%)	DAC (%)	AVC/AIT (%)	FA (%)	Marca-passo (%)	NYHA III/IV (%)
Agasthi, 2020	954	80,9 ± 8,7	392 (41)	8,2 ± 5,2	810 (85)	337 (35)	226 (27)‡	91 (9)	410 (43)	149 (16)	720 (75)
(16)											
Amat-santos, 2018	813	81 ± 7	522 (64)	6,9 ± 5,1	660 (82)	306 (38)	327 (41)	nr	201 (27)	nr	431 (72)
(5)											
Barbanti, 2015	518	81,5 ± 8,4	233 (45)	8,3 ± 5,2	402 (78)	156 (30)	173 (33)‡	76 (15)	198 (38)	86 (17)	449 (87)
(17)											
Barvalia, 2017	460	81,7 ± 8	251 (55)	7,6 ± 4,8	426 (97)	185 (40)	384 (84)	nr	nr	nr	nr
(18)											
Gotzmann, 2011	145	79,1 ± 6,4	nr	EuroSCORE logístico†:	127 (88)	nr	nr	nr	nr	nr	138 (95)
(19)				21 ± 16,2							
Hutter, 2013	268	80,9 ± 6,5	167 (62)	6,3 ± 4,2	nr	nr	142 (53)	36 (13)	62 (23)	nr	268 (100)
(20)											
Kjonas, 2019	218	81,8 ± 7,1	98 (45)	5,6 ± 4,0	148 (68)	62 (28)	82 (38)‡	52 (24)	100 (46)	nr	187 (86)
(21)											
Lindman, 2015	507	84,6 ± 8,5	253 (50)	10,5 ± 5,5	458 (90)	178 (35)	322 (63)	nr	186 (37)	96 (19)	242 (48)§
(22)											
McCarthy, 2018	34.576	81,7 ± 8,8	16.844 (49)	8,3 ± 6,0	30.737 (89)	12.842 (37)	23.873 (69)	4.240 (12)	14.199 (41)	5.702 (16)	28.129 (81)
(6)											
Medvedofsky, 2020	334	83 ± 8,0	197 (59)	9,2 ± 5	314 (94)	110 (33)	63 (19)‡	41 (13)	0 (0)	0 (0)	283 (88)
(12)											
Omar, 2020	174	83,5 [78,4–88,0]	84 (48)	7,3 [4,7–13,6]	159 (91)	59 (34)	80 (46)‡	18 (10)	75 (43)	nr	158 (91)
(23)											
Schwartz, 2016	519	85,6 ± 6	296 (57)	EuroSCORE†:	452 (87)	182 (35)	311 (60)	nr	85 (16)	54 (10)	483 (93)
(24)				20,5 ± 14							
Schymik, 2015	2.688	81,4 ± 6,6	1.550 (58)	7,9 ± 6,6	2.175 (81)	791 (29)	1.188 (44)	345 (13)	685 (26)	304 (11)	2.057 (77)
(25)											
Sultan, 2018	457	84,0 [52,0–97,0]	222 (49)	7,8 (1,0-38,0)	408 (89)	177 (39)	171 (37)‡	nr	206 (45)	nr	444 (97)
(26)											
Veulemans, 2019	874	80,5 ± 6,1	469 (54)	6,8 ± 6,6	819 (94)	283 (32)	645 (74)	174 (20)	285 (33)	112 (13)	616 (70)
(27)											
Wendler, 2017	1.946	81,5 ± 6,7	934 (48)	EuroSCORE logístico†:	1.591 (82)	575 (29)	1.002 (51)	376 (19)	424 (23)	230 (12)	1.378 (73)
(28)				13,96 [8,97, 22,78] – TF							
				17,83 [11,40, 29,25] – não TF							
Worku, 2018	369	86,4	193 (52)	9,8	325 (88)	122 (33)	74 (20)‡	82 (22)	142 (39)	67 (18)	234 (63)
(29)											

Os valores são média ± DP ou mediana (mín-máx) ou [intervalo interquartil] ou n (%). AIT: ataque isquêmico transitório; AVC: acidente vascular cerebral; DAC: doença arterial coronariana; DM: diabetes mellitus; EuroSCORE: European System for Cardiac Operative Risk Evaluation; FA: fibrilação atrial; HT: hipertensão; N°.: número; nr: não relatado; NYHA: New York Heart Association; ref.: referência; STS: Society of Thoracic Surgeons; TAVR: substituição da valva aórtica transcateter; TF: acesso transfemoral; † STS e EuroSCORE são algoritmos baseados na presença de doenças coexistentes para prever a mortalidade operatória em 30 dias. ‡ Caso não claramente declarado, consideramos como evidência de DAC os pacientes com infarto do miocárdio (IM) prévio. § Incluiu apenas pacientes NYHA IV.

**Tabela 3:** – Características ecocardiográficas dos pacientes incluídos

Primeiro autor, ano		Gradiente	AVA		IM	
(N°. de ref.)	FEVE média (%)	médio (mmHg)	média (cm^2^)	PSAP	mod/grave (%)	Disfunção do VD (%)
Agasthi, 2020	57,1 ± 13,1	43,2 ± 13,6	0,87 ± 0,33	42,3 ± 14,4†	46 (5)	nr
(16)						
Amat-santos, 2018	60 [52-70] – IT < 2	47 [39-56] – IT < 2	0,62 [0,5-0,8] – IT < 2	47,2 ± 16,8 – IT < 2	303 (40)	ESPAT:
(5)	60 [50-65] – IT ≥ 2	44 [36-59] – IT ≥ 2	0,64 [0,5-0,8] – IT ≥ 2	49,8 ± 16,6 – IT ≥ 2		21 [19-23] – IT < 2
						20 [17-22] – IT ≥ 2
Barbanti, 2015	53,9 ± 13,9	42,2 ± 16,3	0,7 ± 0,4	43,7 ± 17,8	208 (40)	DDGVD:
(17)						39,9 ± 7,3
Barvalia, 2017	50,9 ± 14,9	47,6 ± 15,5	0,67 ± 0,24	nr	80 (17)	nr
(18)						
Gotzmann, 2011	55,8 ± 12,2	46,6 ± 13,7	nr	91 (63) – PSAP > 25 mmHg	83 (57)	nr
(19)						
Hutter, 2013	44 (16) – FEVE < 35%	48,7 ± 16,7	0,64 ± 0,18	62 (23) – PSAP > 60	60 (22)	45 (17)
(20)						
Kjonas, 2019	110 (50) – FEVE ≥ 50%	51,6 ± 14,8	0,63 ± 0,2	21 (10) – PSAP > 60	45 (21)	ESPAT:
(21)	79 (36) – FEVE 31-49%					1,6 ± 0,5
	23 (10) – FEVE ≤ 30%					
Lindman, 2015	51,2 ± 12,6	45,5 ± 13,7	0,34 ± 0,09*	40 (32-52) – ausente/leve IT	147 (29)	162 (34)
(22)				44 (35-58) – IT mod		
				43 (30-52) – IT grave		
McCarthy, 2018	53,2 ± 14,1	44,2 ± 15,0	nr	46,2 ± 15,0†	10183 (29)	nr
(6)						
Medvedofsky, 2020	53 ± 14	49 ± 13	0,44 ± 0,09	45 ± 16	4 (1)	63 (19)
(12)						
Omar, 2020	57,5 [43–65]	42 ± 15	0,69 ± 0,2	46,0 ± 15,3†	nr	nr
(23)						
Schwartz, 2016	56,3 ± 9	46,9 ± 15	0,71 ± 0,18	42,5 ± 15	109 (21)	84 (16)
(24)						
Schymik, 2015	54,4 ± 12,5	47,6 ± 16,2	0,7 ± 0,2	44,9 ± 14,9	519 (20)	nr
(25)						
Sultan, 2018	53,9 ± 13,4	48,0 ± 15,4	0,63 ± 0,18	44,1 ± 16,8	55 (12)	DDGVD < 16:
(26)						139 (30)
Veulemans, 2019	51,4 ± 12,5	37,0 ± 16,4	0,7 ± 0,2	510 (58) – PSAP ≥ 25 mmHg	155 (18)	nr
(27)						
Wendler, 2017	100 (6) – FEVE < 30%	44,1 ± 16,0	0,73 ± 0,210	nr	249 (14)	nr
(28)						
Worku, 2018	51,6	45,6	0,7	59,4	78 (21)	31 (8)
(29)						

Os valores são média ± DP ou mediana (mín-máx) ou [intervalo interquartil] ou n (%). AVA: área da valva aórtica; DDGVD: diâmetro diastólico final do ventrículo direito; ESPAT: excursão sistólica do plano anular tricúspide; FEVE: fração de ejeção do ventrículo esquerdo; mod: insuficiência tricúspide moderada; nr: não relatado; PSAP: pressão sistólica da artéria pulmonar; ref.: referência; IM: insuficiência mitral; IT: insuficiência tricúspide; VD: ventrículo direito. * Área da valva aórtica indexada à superfície corporal. † Na ausência da PSAP, foi relatada a pressão sistólica do VD.

### Estimativas de risco e avaliações de viés

A maioria dos estudos relatou comparações padrão (IT ausente/leve vs. moderada/grave) para a mortalidade por todas as causas. Em estudos com seguimento de curto prazo, as análises de RC não ajustada foram as mais relatadas, enquanto as comparações ajustadas por RR foram relatadas principalmente por estudos com seguimento de médio prazo. Embora altamente variáveis entre os estudos, as covariáveis clínicas e ecocardiográficas [idade, sexo, STS/EuroSCORE, hipertensão, diabetes, fibrilação atrial (FA), classe funcional da New York Heart Association (NYHA), FEVE, IM e pressão sistólica da artéria pulmonar (PSAP)] foram incluídas nos modelos ( Tabela complementar 1 ). O risco geral de viés foi baixo ou moderado em todos os estudos, com exceção de um^5^ (Kappa = 0.72 [95% CI, 0.54-0.89]) ( Tabela complementar 2 ).

### Análise primária: IT ausente/leve vs. IT moderada/grave

**Figura 2:**
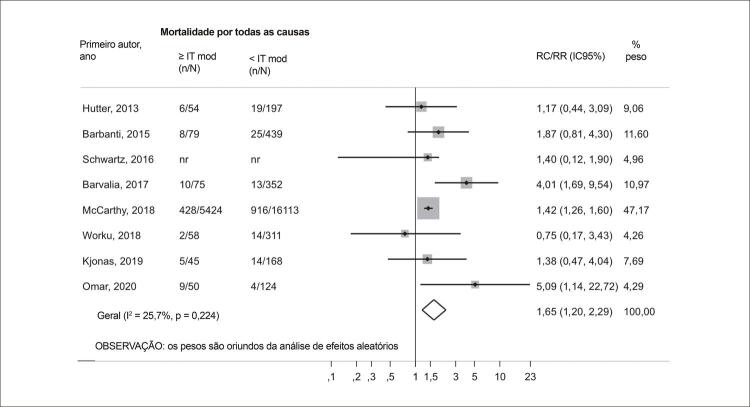
– Gráfico de floresta comparando a mortalidade por todas as causas (30 dias) entre pacientes com graus basais de IT ausente/leve e moderada/grave. IC: intervalo de confiança; mod: insuficiência tricúspide moderada; RC: razão de chances; RR: razão de risco; IT: insuficiência tricúspide.

Aos 30 dias após a TAVR, a IT moderada/grave foi associada a um risco aumentado de mortalidade por todas as causas quando comparada a IT ausente/leve (RR 1,65; IC95% 1,20-2,29; I^2^ = 25,7%; p = 0,224). Após um seguimento médio de 1,2 ano, a análise agrupada de 14 estudos também revelou que graus mais altos de IT foram associados a um pior prognóstico (RR 1,56; IC95% 1,31-1,84; I^2^ = 44,1%; p = 0,039) ( Figuras 2 e 3 ).

**Figura 3:**
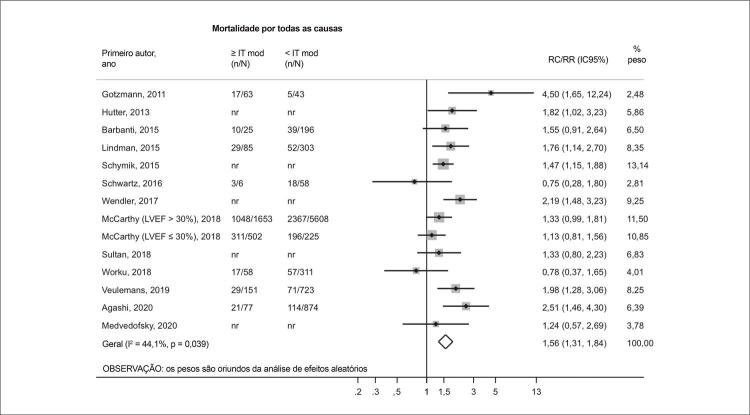
– Gráfico de floresta comparando a mortalidade por todas as causas (1,2 ano) entre pacientes com graus basais de IT ausente/leve e moderada/grave. FEVE: fração de ejeção do ventrículo esquerdo; IC: intervalo de confiança; mod: insuficiência tricúspide moderada; RC: razão de chances; RR: razão de risco; IT: insuficiência tricúspide.

Na análise de sensibilidade *leave-one-out* , as taxas de risco variaram de 1,20-3,0 (curto prazo) e 1,26-1,92 (médio prazo), indicando que a estimativa agrupada era robusta e não influenciada por um único estudo. A análise de subgrupo mostrou menos heterogeneidade [I^2^ = 0%; p = 0,489 (não ajustado) e I^2^ = 39,6%; p = 0,094 (ajustado)] quando os estudos foram agrupados de acordo com estimativas de risco univariadas/multivariadas. A análise de metarregressão revelou que a proporção de pacientes com IT significativa em cada estudo não alterou a associação entre a IT e a mortalidade por todas as causas (p = 0,676). Essas análises revelaram a ausência de viés de publicação, ou seja, gráficos de funil simétricos e p > 0,05 para todos os testes de regressão linear de Egger ( Figuras complementares 1–3 ).

### Análise secundária: mortalidade e gravidade da IT

**Figura 4:**
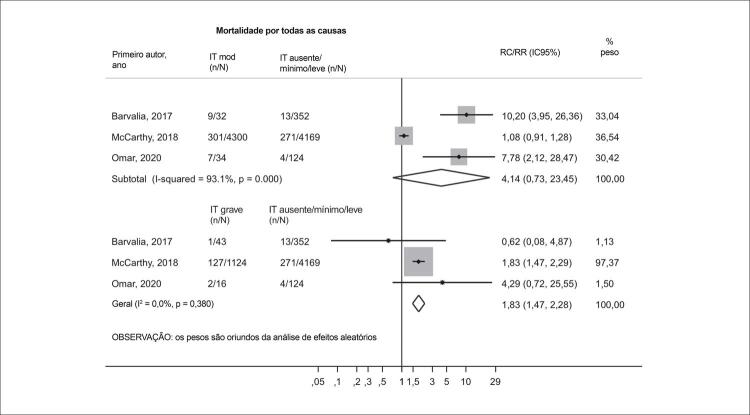
– Gráfico de floresta comparando a mortalidade por todas as causas (30 dias) em pacientes com graus crescentes de IT. IC: intervalo de confiança; mod: insuficiência tricúspide moderada; RC: razão de chances; RR: razão de risco; IT: insuficiência tricúspide.

No curto prazo, não observamos diferenças estatisticamente significativas na mortalidade por todas as causas entre pacientes com IT moderada e aqueles com IT ausente/leve (RR 4,14; IC95% 0,73-23,45), apesar da alta heterogeneidade (I^2^ = 93,1%; p < 0,001). No entanto, a IT grave foi associada a um aumento de 83% na mortalidade quando comparada à IT ausente/leve (RR 1,83; IC95% 1,47-2,28; I^2^ = 0%; p = 0,380) ( Figura 4 ).

**Figura 5:**
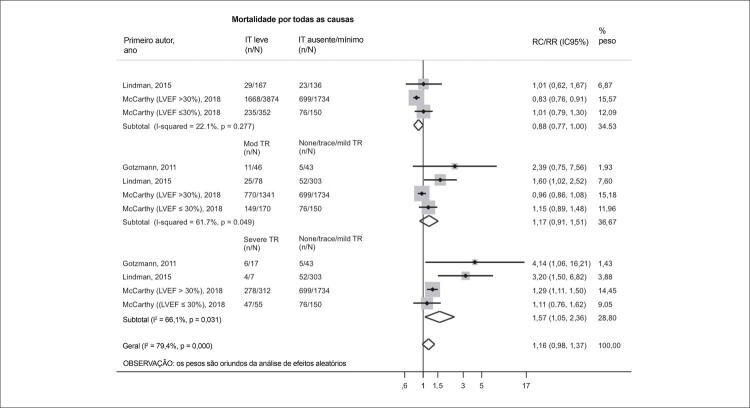
– Gráfico de floresta comparando a mortalidade por todas as causas (318 dias) em pacientes com graus crescentes de IT. FEVE: fração de ejeção do ventrículo esquerdo; IC: intervalo de confiança; mod: insuficiência tricúspide moderada; RC: razão de chances; RR: razão de risco; IT: insuficiência tricúspide.

A médio prazo (média de 318 dias), ao comparar pacientes com IT ausente/mínimo com aqueles com IT leve (RR 0,88; IC95% 0,77-1,00) e IT moderada (RR 1,17; IC95% 0,91-1,51), não foram observadas diferenças nas taxas de risco de mortalidade. Porém, a IT grave foi associada a um risco significativamente aumentado de mortalidade por todas as causas quando comparada à IT ausente/mínimo (RR 1,57; IC95% 1,05-2,36), embora com heterogeneidade moderada (I^2^ = 66,1%; p = 0,031) ( Figura 5 ).

### Outros desfechos

Os detalhes sobre a mortalidade cardiovascular e re-hospitalização por ICa são mostrados na Tabela complementar 3 . Em geral, as estimativas de risco mais alto de desfechos foram observadas em indivíduos com graus de IT mais altos.

### Análises adicionais: alterações na gravidade da IT após a TAVR

Treze estudos^17 , 20 , 22 - 24 , 29 - 36^ somando 709 pacientes relataram graus de IT pré- e pós-TAVR. Os detalhes dos estudos e as características dos pacientes são mostrados na Tabela complementar 4. Com exceção de dois estudos^31 , 35^ (que avaliaram apenas graus de IT graves), todos os outros relataram mudanças nos graus de IT moderada/grave pós-TAVR. Oito estudos reavaliaram os graus de IT no curto prazo (até 30 dias), e oito estudos revisitaram os graus de IT após 30 dias (média = 12,5 meses).

**Figura 6:**
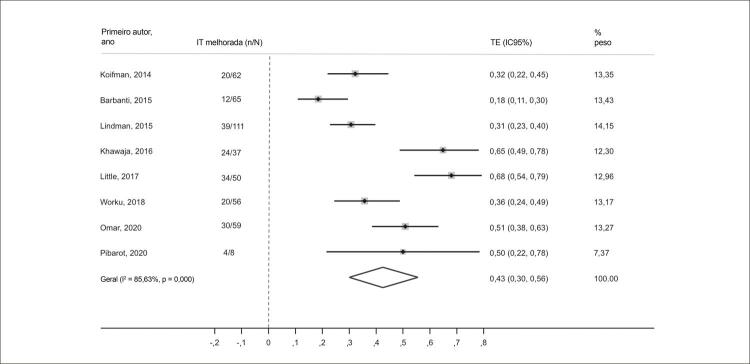
– Alterações nos graus de IT moderada/grave 30 dias pós-TAVR. IC: intervalo de confiança; IT: insuficiência tricúspide; TE: tamanho do efeito.

No dia 30 após a TAVR, a gravidade da IT diminuiu em pelo menos um grau em 43% dos pacientes (IC95% 0,30-0,56; I^2^ = 85,6%; p < 0,001). Em um seguimento médio de 12,5 meses, 44% dos pacientes apresentaram melhora nos graus de IT após a TAVR (IC95% 0,35-0,52; I^2^ = 61,6%; p = 0,01) ( Figuras 6 e 7 ). As análises de metarregressão revelaram que as melhorias nos graus da IT (a curto e médio prazo) não foram influenciadas pela proporção de pacientes com FA ou disfunção do ventrículo direito (VD)ou pelos valores de PSAP (p > 0,05 para todos).

**Figura 7:**
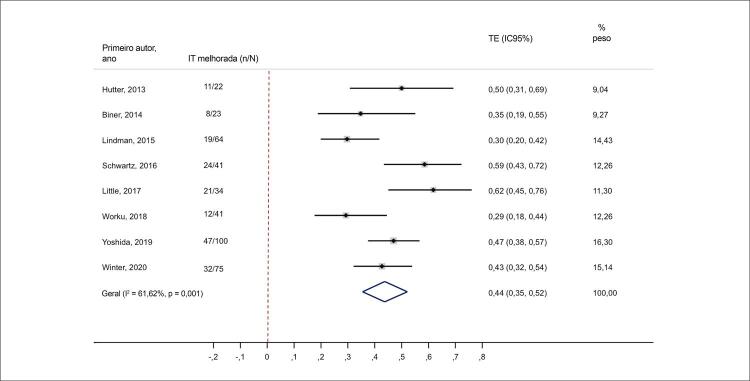
– Alterações nos graus de IT moderada/grave em médio prazo pós-TAVR. IC: intervalo de confiança; IT: insuficiência tricúspide; TE: tamanho do efeito.

**Figura 8:**
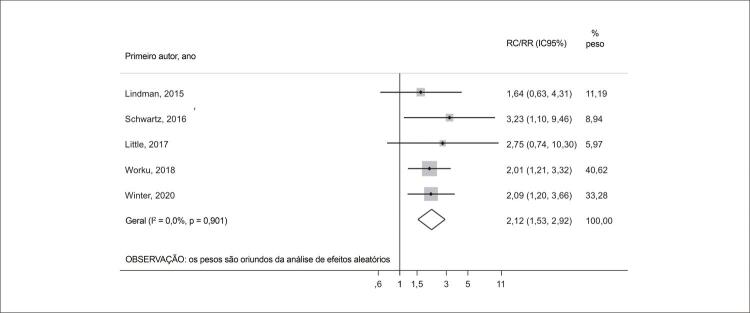
– Gráfico de floresta comparando a mortalidade por todas as causas entre pacientes com persistência e melhora dos graus de IT pós-TAVR. IC: intervalo de confiança; RC: razão de chances; RR: razão de risco.

Na análise agrupada, a persistência dos graus de IT moderada/grave após um seguimento médio de 21 meses pós-TAVR foi associada à mortalidade por todas as causas (RR 2,12; IC95% 1,53-2,92; I^2^ = 0%, p = 0,901) ( Figura 8 ). Não foi detectada nenhuma alteração significativa no tamanho de efeito geral após a realização de uma análise de sensibilidade leave-one-out, e não foi registrada nenhuma evidência de viés de publicação entre os estudos ( Figuras complementares 4 e 5 ).

## Discussão

Esta metanálise com 23 estudos incluindo mais de 45.000 pacientes e avaliando a associação entre IT e desfechos clínicos após a TAVR tem três achados principais. Primeiro, a IT moderada ou grave na linha de base foi associada ao aumento da mortalidade por todas as causas, tanto em 30 dias quanto no médio prazo (1,2 ano); segundo, observou-se um gradiente entre a gravidade da IT e a mortalidade. Pacientes com IT grave tiveram pelo menos 57% de aumento no risco de morte no médio prazo (318 dias) quando comparados àqueles com IT ausente/mínimo. Em terceiro lugar, após a TAVR, a gravidade da IT melhorou em pelo menos um grau em > 40% dos pacientes. Pacientes sem melhora quanto à gravidade da IT após o procedimento apresentaram piores desfechos. Nossos resultados confirmam os principais achados de metanálises semelhantes nesse campo.^37 - 39^ De forma exclusiva, além de incluirmos dados de um registro recente e amplo da STS,^6^ avaliamos a associação entre alteração do grau de IT e mortalidade subsequente e demonstramos um gradiente com a maior mortalidade observada entre os pacientes com IT grave. Por fim, também analisamos as alterações na gravidade da IT após a TAVR e a associação entre IT significativa persistente e a sobrevida.

A relação entre IT concomitante e o prognóstico recebeu pouca atenção em estudos tradicionais de TAVR, com achados controversos sendo descritos. Embora vários relatos^18 , 19 , 28^ tenham sugerido aumento da mortalidade quando IT significativa foi detectada pré-procedimento, outros observaram que essa associação não era mais significativa após o ajuste multivariado^20 , 21 , 24 , 25 , 29^ ou que existia apenas quando a IT significativa persistia após a TAVR,^22 , 24 , 29 , 36^ independentemente da gravidade basal da IT.^22^ Ainda que os pacientes com IT moderada/grave tenham apresentado mais comorbidades e maiores riscos,^6 , 17 , 20 , 22 , 24 , 29^ depois de agrupar os resultados de todos os ajustes multivariados, na nossa metanálise, a presença de IT significativa permaneceu relacionada a um pior prognóstico após a TAVR.

Ainda não está claro se a IT representa um marcador substituto de doença tardia ou um fator de risco em si. A relação independente entre a IT e um pior prognóstico após a TAVR foi relatada em cenários de FEVE maior que 30 a 40%^6 , 17^ ou com graus menores de IM,^5 , 22^ possivelmente apontando para mecanismos orgânicos de IT não passíveis de AVR.^29^ De forma controversa, uma análise de subgrupo feita por Gotzmann et al.^19^ sugeriu que a etiologia subjacente da IT (orgânica ou funcional) não teve impacto incremental na mortalidade por todas as causas pós-TAVR. Embora não possamos garantir, uma vez que 44% dos pacientes com IT melhoraram em pelo menos um grau após a TAVR, é razoável supor que, na nossa metanálise, um número significativo de etiologias da IT foi secundário. Além disso, observamos mais do que o dobro de risco de mortalidade por todas as causas em pacientes com IT moderada/grave persistente após a TAVR.

Hipertensão pulmonar sustentada,^24 , 29^ FA,^24 , 29^ diâmetro do anel tricúspide^24^ e dilatação do VD^29^ são os principais fatores associados à não melhora da IT após a TAVR. A relação com a disfunção do VD é controversa,^24 , 29^ porque, talvez, a dilatação do VD reflita melhor a cronicidade e a gravidade da sobrecarga do VD do que a função ventricular.^22^ Talvez, mais importante do que a quantificação isolada dos parâmetros, seja a avaliação conjunta do acoplamento ventrículo direito-artéria pulmonar, integrando o desempenho da unidade à direita.^26 , 40^ Em nossa análise de metarregressão, as melhorias nos graus de IT não foram influenciadas pela proporção de pacientes com FA, disfunção do VD ou pelos valores da PSAP, mas vários critérios e métodos foram usados para definir essas variáveis.

Reconhecer a associação da IT e o pior prognóstico pós-TAVR ajuda no manejo clínico e influencia as decisões da equipe de cardiologia. A seleção apropriada do paciente é crucial para o sucesso do procedimento^41 - 43^ e, até o momento, além da FEVE reduzida, não há recomendações sobre a importância das consequências cardíacas anatômicas ou funcionais da EA como um componente do algoritmo de decisão para AVR.^3^ Nossos achados reforçam a necessidade de uma avaliação cuidadosa da IT antes da TAVR, incluindo melhores estratificações de risco que possam identificar subgrupos de pacientes nos quais seja esperado que a evolução clínica pós-TAVR seja pior. Essas recomendações evitam a futilidade relacionada à TAVR, a qual pode influenciar tanto a qualidade de vida quanto os custos com saúde.^5^

Como o uso da TAVR está aumentando rapidamente, são obrigatórias as avaliações dos benefícios antecipados de tratamentos cirúrgicos para doenças multivalvares.^6 , 22 , 24^ Para os candidatos à cirurgia, a inclusão de um reparo tricúspide à AVR cirúrgica (SAVR) de coração aberto pode levar a melhores desfechos do que a TAVR sem tratamento da IT ou posterior reparo cirúrgico isolado da IT.^5 , 6 , 22^ É interessante notar que, embora as diretrizes atuais forneçam recomendações de classe I para anuloplastia da valva tricúspide nesse cenário,^41 - 43^ os desfechos da cirurgia tricúspide podem não ser os ideais.^44^ Além disso, para a IT secundária à disfunção do VD, o reparo da valva tricúspide pode, potencialmente, precipitar a insuficiência VD grave secundária ao aumento da pós-carga do VD e, nesse caso, a TAVR pode ser preferível, em detrimento à SAVR.^29^ Por todos os motivos mencionados, a estratificação de risco adequada, a avaliação cuidadosa da valva tricúspide e os fatores associados que podem predizer a persistência de IT corroboram a decisão por TAVR em relação à SAVR e podem levar a estratégias alternativas de tratamento transcateter da IT, que devem ser testadas em estudos prospectivos randomizados.

### Limitações do estudo

Em primeiro lugar, como este estudo se tratou de uma revisão sistemática e metanálise de estudos não randomizados, ele trouxe consigo as limitações inerentes da pesquisa observacional, apesar do rigor metodológico robusto e das análises de sensibilidade. Em segundo lugar, o número relativamente baixo de estudos limitou a análise de desfechos diferentes da mortalidade por todas as causas. Em terceiro lugar, a análise do grau de IT foi totalmente dependente de ecocardiogramas e, embora a maioria dos estudos realizasse avaliações de IT de acordo com as diretrizes padrão, alguns publicaram dados relatados no local.^6 , 25^ Em quarto lugar, reunir os graus de IT moderada e grave em um só grupo pode ter reunido pacientes com prognósticos diferentes. Por isso, avaliamos o risco incremental de cada grau de IT adicional e mostramos uma relação do tipo “dose-resposta” com a sobrevida. Além disso, é interessante ressaltar que, recentemente, foi proposta uma nova classificação para a IT,^45^ pois foi demonstrado que, mesmo em pacientes com IT significante, a mortalidade aumenta à medida que a IT se torna maciça ou torrencial.^46^ Portanto, em estudos futuros, também deve ser realizada uma análise que reestratifique pacientes com IT grave.

Apesar das limitações, a ampla coorte do nosso estudo e os achados robustos sugerem a necessidade de futuros ensaios clínicos randomizados dedicados a avaliar o impacto da IT no prognóstico da TAVR, incluindo a investigação de fatores relacionados à persistência da IT após a TAVR, o que, conforme demonstrado aqui, está associada a desfechos adversos.

## Conclusões

A presença de IT significativa pré-TAVR está associada a maior mortalidade. Ainda que, geralmente, a gravidade da IT melhore pós-TAVR, a persistência de IT significativa está fortemente associada ao aumento da mortalidade. Nossos achados destacam a importância da IT pré- e pós-TAVR e podem ajudar a identificar pacientes que possam se beneficiar de uma vigilância mais cuidadosa nesse cenário clínico.

## *Material suplementar

Para informação adicional, por favor, clique aqui


