# Supplementary material for: Insuficiência Tricúspide e Mortalidade em Pacientes Submetidos à Troca da Valva Aórtica Transcateter: Uma Revisão Sistemática e Metanálise
Source: Arq Bras Cardiol. 2023 Jul 27;120(7):e20220319. [Article in Portuguese] doi: 10.36660/abc.20220319 (PMC10382153; doi:10.36660/abc.20220319)
Supplement: Supplementary file 1 [file 2022-0319-supplemental-material.pdf]

SUPPLEMENTAL MATERIAL

1 - Details of search strategy:

PubMed

(((("transcatheter aortic valve replacement"[MeSH Terms] OR ("transcatheter"[All Fields] AND "aortic"[All Fields] AND "valve"[All Fields] AND "replacement"[All Fields]) OR "transcatheter aortic valve replacement"[All Fields] OR ("transcatheter"[All Fields] AND "aortic"[All Fields] AND "valve"[All Fields] AND "implantation"[All Fields]) OR "transcatheter aortic valve implantation"[All Fields]) OR ("transcatheter aortic valve replacement"[MeSH Terms] OR ("transcatheter"[All Fields] AND "aortic"[All Fields] AND "valve"[All Fields] AND "replacement"[All Fields]) OR "transcatheter aortic valve replacement"[All Fields])) OR tavi[All Fields]) OR tavr[All Fields]) AND ((((("tricuspid valve insufficiency"[MeSH Terms] OR ("tricuspid"[All Fields] AND "valve"[All Fields] AND "insufficiency"[All Fields]) OR "tricuspid valve insufficiency"[All Fields] OR ("tricuspid"[All Fields] AND "regurgitation"[All Fields]) OR "tricuspid regurgitation"[All Fields]) OR ("tricuspid valve insufficiency"[MeSH Terms] OR ("tricuspid"[All Fields] AND "valve"[All Fields] AND "insufficiency"[All Fields]) OR "tricuspid valve insufficiency"[All Fields] OR ("tricuspid"[All Fields] AND "valve"[All Fields] AND "regurgitation"[All Fields]) OR "tricuspid valve regurgitation"[All Fields])) OR ("tricuspid valve insufficiency"[MeSH Terms] OR ("tricuspid"[All Fields] AND "valve"[All Fields] AND "insufficiency"[All Fields]) OR "tricuspid valve insufficiency"[All Fields] OR ("tricuspid"[All Fields] AND "incompetence"[All Fields]) OR "tricuspid incompetence"[All Fields])) OR ("tricuspid valve insufficiency"[MeSH Terms] OR ("tricuspid"[All Fields] AND "valve"[All Fields] AND "insufficiency"[All Fields]) OR "tricuspid valve insufficiency"[All Fields] OR ("tricuspid"[All Fields] AND "valve"[All Fields] AND "incompetence"[All Fields]) OR "tricuspid valve incompetence"[All Fields])) OR ("tricuspid valve insufficiency"[MeSH Terms] OR ("tricuspid"[All Fields] AND "valve"[All Fields] AND "insufficiency"[All Fields]) OR "tricuspid valve insufficiency"[All Fields]))) AND ((((((("prognosis"[MeSH Terms] OR "prognosis"[All Fields]) OR ("prognosis"[MeSH Terms] OR "prognosis"[All Fields] OR "prognoses"[All Fields])) OR ("prognosis"[MeSH Terms] OR "prognosis"[All Fields] OR ("prognostic"[All Fields] AND "factors"[All Fields]) OR "prognostic factors"[All Fields])) OR ((((("mortality"[Subheading] OR "mortality"[All Fields] OR "mortality"[MeSH Terms]) OR ("mortality"[MeSH Terms] OR "mortality"[All Fields] OR "mortalities"[All Fields])) OR ("mortality"[MeSH Terms] OR "mortality"[All Fields] OR ("mortality"[All Fields] AND "determinants"[All Fields]) OR "mortality determinants"[All Fields])) OR ("mortality"[Subheading] OR "mortality"[All Fields] OR ("death"[All Fields] AND "rate"[All Fields]) OR "death rate"[All Fields] OR "mortality"[MeSH Terms] OR ("death"[All Fields] AND "rate"[All Fields]) OR "death rate"[All Fields])) OR ("mortality"[MeSH Terms] OR "mortality"[All Fields] OR ("mortality"[All Fields] AND "rate"[All Fields]) OR "mortality rate"[All Fields]))) OR ("death"[MeSH Terms] OR "death"[All Fields])) OR (("hospitalisation"[All Fields] OR "hospitalization"[MeSH Terms] OR "hospitalization"[All Fields]) OR ("hospitalization"[MeSH Terms] OR

"hospitalization"[All Fields] OR "hospitalizations"[All Fields]))) OR ((((("heart failure"[MeSH Terms] OR ("heart"[All Fields] AND "failure"[All Fields]) OR "heart failure"[All Fields]) OR ("heart failure"[MeSH Terms] OR ("heart"[All Fields] AND "failure"[All Fields]) OR "heart failure"[All Fields] OR ("cardiac"[All Fields] AND "failure"[All Fields]) OR "cardiac failure"[All Fields])) OR ("heart failure"[MeSH Terms] OR ("heart"[All Fields] AND "failure"[All Fields]) OR "heart failure"[All Fields] OR ("heart"[All Fields] AND "decompensation"[All Fields]) OR "heart decompensation"[All Fields])) OR ("heart failure"[MeSH Terms] OR ("heart"[All Fields] AND "failure"[All Fields]) OR "heart failure"[All Fields] OR ("myocardial"[All Fields] AND "failure"[All Fields]) OR "myocardial failure"[All Fields])) OR ("heart failure"[MeSH Terms] OR ("heart"[All Fields] AND "failure"[All Fields]) OR "heart failure"[All Fields] OR ("congestive"[All Fields] AND "heart"[All Fields] AND "failure"[All Fields]) OR "congestive heart failure"[All Fields])))

1.1.1 SCOPUS

(TITLE-ABS (*tavr*) OR TITLE-ABS (*tavi*) OR TITLE-ABS (*transcatheter* AND *aortic* AND *valve**)) AND TITLE-ABS-KEY (*tricuspid**) AND (TITLE-ABS-KEY (*death*) OR TITLE-ABS-KEY (*prognosis*) OR TITLE-ABS-KEY (*mortality*) OR TITLE-ABS-KEY (*cardiac* AND *death*) OR TITLE-ABS-KEY (*hospitalization*) OR TITLE-ABS-KEY (*heart* AND *failure*)) AND (LIMIT-TO (SUBJAREA , *"MEDI"*)) AND (LIMIT-TO (DOCTYPE , *"ar"*))

LILACS

Transcatheter Aortic Valve Replacement AND tricuspid valve

Web of Science

((ALL=(TAVI OR TAVR OR transcatheter aortic valve implantation OR transcateter aortic valve replacement)) AND (ALL=(tricuspid regurgitation OR tricuspid valve insufficiency OR tricuspid incompetence)) AND (ALL=(death OR prognosis OR mortality OR cardiac death OR hospitalization OR heart failure)))
Índices=SCI-EXPANDED, SSCI, A&HCI, CPCI-S, CPCI-SSH, ESCI Tempo estipulado=Todos os anos

EMBASE

('transcatheter aortic valve implantation'/exp OR 'tavi' OR 'percutaneous aortic valve implantation' OR 'percutaneous aortic valve replacement' OR 'trans-arterial aortic

valve implantation' OR 'trans-arterial aortic valve replacement' OR 'trans-catheter aortic valve implantation' OR 'trans-catheter aortic valve replacement' OR 'trans-cutaneous aortic valve implantation' OR 'trans-cutaneous aortic valve replacement' OR 'trans-femoral aortic valve implantation' OR 'trans-femoral aortic valve replacement' OR 'transarterial aortic valve implantation' OR 'transarterial aortic valve replacement' OR 'transcatheter aortic valve implantation' OR 'transcatheter aortic valve replacement' OR 'transcutaneous aortic valve implantation' OR 'transcutaneous aortic valve replacement' OR 'transfemoral aortic valve implantation' OR 'transfemoral aortic valve replacement' OR 'transapical aortic valve replacement' OR 'transapical aortic valve implantation' OR 'trans-apical aortic valve implantation' OR 'trans-apical aortic valve replacement' OR 'percutaneous aortic valve'/exp OR 'acurate ta' OR 'acurate tf' OR 'centera' OR 'corevalve revalving' OR 'corevalve system' OR 'cribier edwards valve' OR 'directflow' OR 'edwards sapien 3' OR 'edwards sapien xt' OR 'edwards sapien valve' OR 'engager (device)' OR 'inovare valve' OR 'jenavalve' OR 'lotus edge' OR 'lotus valve system' OR 'portico' OR 'percutaneous aortic valve' OR 'percutaneous aortic valve prosthesis' OR 'transcatheter aortic valve prosthesis') AND ('tricuspid valve regurgitation'/exp OR 'heart valve insufficiency, tricuspid' OR 'heart valve regurgitation, tricuspid' OR 'regurgitation, right atrioventricular heart valve' OR 'right atrioventricular cardiac regurgitation' OR 'right atrioventricular cardiac valve insufficiency' OR 'right atrioventricular cardiac valvular insuffiency' OR 'right atrioventricular cardiac valvular regurgitation' OR 'right atrioventricular heart valve insufficiency' OR 'right atrioventricular heart valve regurgitation' OR 'right atrioventricular heart valvular regurgitation' OR 'right atrioventricular valve insufficiency' OR 'right atrioventricular valve regurgitation' OR 'right atrioventricular valvular insufficiency' OR 'right atrioventricular valvular regurgitation' OR 'tricuspid cardiac valve insufficiency' OR 'tricuspid cardiac valve regurgitation' OR 'tricuspid cardiac valvular insufficiency' OR 'tricuspid cardiac valvular regurgitation' OR 'tricuspid heart valve insufficiency' OR 'tricuspid heart valve regurgitation' OR 'tricuspid heart valvular insufficiency' OR 'tricuspid heart valvular regurgitation' OR 'tricuspid insufficiency' OR 'tricuspid regurgitation' OR 'tricuspid valve insufficiency' OR 'tricuspid valve regurgitation' OR 'tricuspid valvular insufficiency' OR 'tricuspid valvular regurgitation' OR 'tricuspidal insufficiency') AND ('mortality'/exp OR 'excess mortality' OR 'mortality' OR 'mortality model' OR 'death'/exp OR 'death' OR 'mors' OR 'prognosis'/exp OR 'prognosis' OR 'heart death'/exp OR 'cardiac death' OR 'heart

death' OR 'hospitalization'/exp OR 'hospital stay' OR 'hospitalization' OR 'short stay hospitalization' OR 'heart failure'/exp OR 'backward failure, heart' OR 'cardiac backward failure' OR 'cardiac decompensation' OR 'cardiac failure' OR 'cardiac incompetence' OR 'cardiac insufficiency' OR 'cardiac stand still' OR 'cardial decompensation' OR 'cardial insufficiency' OR 'chronic heart failure' OR 'chronic heart insufficiency' OR 'decompensatio cordis' OR 'decompensation, heart' OR 'heart backward failure' OR 'heart decompensation' OR 'heart failure' OR 'heart incompetence' OR 'heart insufficiency' OR 'insufficientia cardis' OR 'myocardial failure' OR 'myocardial insufficiency') AND ([article]/lim OR [article in press]/lim)

2 - Data extraction sheet:
Available online:
https://docs.google.com/forms/d/1z0OEeYVl3EKeQKct0cPAQDfwGehgNkXAP7ZdFnEiVMM/edit

SUPPLEMENTAL TABLES

Table 1 – Risk estimates of each studied included in the meta-analyses

First author, year (Ref. #)	Short term follow-up					Long term follow-up				
	Comparison of TR severity	Risk estimate	HR/OR	Lower 95% CI	Upper 95% CI	Comparison of TR severity	Risk estimate	HR/OR	Lower 95% CI	Upper 95% CI
Agasthi, 2020 (16)	nr					< mod versus ≥ mod	Univariate (OR)	2,51	1,46	4,3
Amat-santos, 2018 (5)	nr					< 2 versus ≥ 2	Multivariate (HR)	18,43	10,19	33,34
Barbanti, 2015 (17)	< mod versus ≥ mod	Univariate (OR)	1,87	0,81	4,3	< mod versus ≥ mod	Multivariate (HR)	1,55	0,91	2,64
Barvalia, 2017 (18)	< mod versus ≥ mod	Univariate (OR)	4,01	1,69	9,54	nr				
	mild versus mod	Univariate (OR)	10,2	3,95	26,36					
	mild versus severe	Univariate (OR)	0,62	0,079	4,87					
Gotzmann, 2011 (19)	nr					< mod versus ≥ mod	Univariate (OR)	4,50	1,652	12,237
						mild versus mod	Univariate (OR)	2,39	0,75	7,56
						mild versus severe	Univariate (OR)	4,14	1,06	16,21
Hutter, 2013 (20)	< mod versus ≥ mod	Univariate (OR)	1,17	0,44	3,09	< mod versus ≥ mod	Univariate (HR)	1,82	1,02	3,23
Kjonas, 2019 (21)	< mod versus ≥ mod	Univariate (OR)	1,38	0,47	4,04	nr				
Lindman, 2015 (22)	nr					< mod versus ≥ mod	Multivariate (HR)	1,76	1,14	2,7
						none/trace versus mild	Univariada (HR)	1,01	0,62	1,67
						mild versus mod	Multivariate (HR)	1,60	1,02	2,52
						mild versus severe	Multivariate (HR)	3,20	1,50	6,82
McCarthy, 2018 (6)	< mod versus ≥ mod	Univariate (OR)	1,42	1,26	1,6					
	none/trace versus mod	Univariate (OR)	1,08	0,91	1,28					
	none/trace versus severe	Univariate (OR)	1,83	1,47	2,29					
- LVEF > 30%						< mod versus ≥ mod	Multivariate (HR)	1,33	0,99	1,81
						none/trace versus mild	Multivariate (HR)	0,83	0,76	0,91
						none/trace versus mod	Multivariate (HR)	0,96	0,86	1,08
						none/trace versus severe	Multivariate (HR)	1,29	1,11	1,5
- LVEF ≤ 30%						< mod versus ≥ mod	Multivariate (HR)	1,13	0,81	1,56
						none/trace versus mild	Multivariate (HR)	1,01	0,79	1,3
						none/trace versus mod	Multivariate (HR)	1,15	0,89	1,48
						none/trace versus severe	Multivariate (HR)	1,11	0,76	1,62
Medvedofsky, 2020 (12)	nr					non severe versus severe	Univariate (HR)	1,24	0,57	2,69
Omar, 2020 (23)	< mod versus ≥ mod	Multivariate	5,09	1,14	22,72	nr				
	mild versus mod	Univariate (OR)	7,78	2,12	28,47					
	mild versus severe	Univariate (OR)	4,29	0,72	25,55					
Schwartz, 2016 (24)	< mod versus ≥ mod	Univariate (OR)	1,4	0,12	1,9	< mod versus ≥ mod	Multivariate (HR)	0,75	0,28	1,8
Schymik, 2015 (25)	nr					< mod versus ≥ mod	Multivariate (HR)	1,47	1,15	1,88
Sultan, 2018 (26)	nr					< mod versus ≥ mod	Multivariate (HR)	1,33	0,8	2,23
Veulemans, 2019 (27)	nr					< mod versus ≥ mod	Multivariate (HR)	1,98	1,28	3,06
Wendler, 2017 (28)	nr					< mod versus ≥ mod	Univariate (HR)	2,19	1,48	3,23
Worku, 2018 (29)	< mod versus ≥ mod	Univariate (OR)	0,75	0,17	3,43	< mod versus ≥ mod	Multivariate (OR)	0,78	0,37	1,65

Legend: CI = confidence interval, HR = hazard ratio, LVEF = left ventricular ejection, mod = moderate tricuspid regurgitation, nr = non reported, OR = odds ratio, Ref. # = reference number, TR = tricuspid regurgitation.

Table 2 – Risk of bias assessment

First autor, year (Ref. #)	Quality (NCO)		
	Selection	Comparability*	Outcome†
Agasthi, 2020 (16)	* * * *	* *	* * *
Amat-santos, 2018 (5)	* * * *		* *
Barbanti, 2015 (17)	* * * *	*	* *
Barvalia, 2017 (18)	* * * *	*	* *
Gotzmann, 2011 (19)	* * * *	* *	*
Hutter, 2013 (20)	* * * *	*	* * *
Kjonas, 2019 (21)	* * * *	*	* *
Lindman, 2015 (22)	* * * *	* *	* * *
McCarthy, 2018 (6)	* * * *	*	* *
Medvedofsky, 2020 (12)	* * * *	*	* *
Omar, 2020 (23)	* * * *	* *	*
Schwartz, 2016 (24)	* * * *	* *	*
Schymik, 2015 (25)	* * * *	* *	* * *
Sultan, 2018 (26)	* * * *	* *	* *
Veulemans, 2019 (27)	* * * *	* *	* *
Wendler, 2017 (28)	* * * *	* *	* * *
Worku, 2018 (29)	* * * *	* *	* *

Legend: NCO = Newcastle Ottawa scale, Ref. # = reference number

This scale used a star system to evaluate studies across three domains: (1) selection of participants, (2) comparability of study groups and, (3) the assessment of outcomes of interest. Available online at:

(http://www.ohri.ca/programs/clinical_epidemiology/nosgen.pdf)

* For the comparability item, it was defined that the study would score in the first item if adjustment of multivariate models including STS or EuroSCORE were

reported; and in the second item if adjustment was performed for at least two clinical variables (among: age, sex, hypertension, diabetes mellitus, coronary artery disease, ischemic transient attack or stroke, atrial fibrillation, pacemaker or NYHA functional status) and two echocardiographic variables (among: LVEF, mean aortic gradient, aortic valve area, pulmonary artery systolic pressure, mitral regurgitation, right ventricle dysfunction).

† For the outcome item, 30 days of follow-up was considered appropriate (for short-term studies) and at least one year follow-up (for long-term studies) was acceptable. Studies also scored if <25% of loss to follow-up was reported.

Table 3 – Risk estimates for other outcomes

First author, year<br>Ref. #	Follow-up	Comparison of TR severity	Risk estimate	HR/OR	Lower 95% CI	Upper 95% CI
**Cardiovascular mortality**						
Barbanti, 2015<br>(17)	30 days	< mod versus ≥ mod	Univariate (OR)	4,17	1,29	13,5
	2 years		Univariate (OR)	3,04	1,36	6,76
Omar, 2020<br>(23)	At discharge	< mod versus ≥ mod	Univariate (RR)	14,67	1,35	159,51
**HF hospitalizations**						
Barbanti, 2015<br>(17)	2 years	< mod versus ≥ mod	Univariate (OR)	1,38	0,68	2,8
McCarthy, 2018<br>(6)						
- LVEF > 30%	1 year	none/trace versus mild	Multivariate (HR)	0,95	0,84	1,07
		none/trace versus mod	Multivariate (HR)	1,09	0,95	1,25
		none/trace versus severe	Multivariate (HR)	1,27	1,04	1,54
- LVEF ≤ 30%		none/trace versus mild	Multivariate (HR)	0,57	0,36	0,91
		none/trace versus mod	Multivariate (HR)	0,84	0,64	1,11
		none/trace versus severe	Multivariate (HR)	0,81	0,63	1,05
**Cardiovascular mortality or HF hospitalizations**						
Schwartz, 2016<br>(24)	1.5 ± 1.17 years	< mod versus ≥ mod	Multivariate (HR)	0,7	0,3	1,5

Legend: CI = confidence interval, HF = heart failure, HR = hazard ratio, LVEF = left ventricular ejection fraction, mod = moderate tricuspid regurgitation, OR = odds ratio, Ref. # = reference number, RR = risk ratio, TR = tricuspid regurgitation.

Table 4 – Main characteristics of studies evaluating changes in TR severity after TAVR

First author, year (Ref. #)	No. of participants urdergoing TAVR	TR severity pre-TAVR (%)	Time which TR severity was reassessed	No. of mod/severe TR reassessed (%)	No. of TR improvement
Barbanti, 2015 (17)	518	< mod = 439 (85)<br>≥ mod = 79 (15)	30 days	65 (82)	12 (18)
Biner, 2014 **(30)**	230	< 2 = 207 (90)<br>≥ 2 = 23 (10)	6 months	23 (100)	8 (35)
Hutter, 2013 (20)	251	< mod = 197 (78)<br>≥ mod = 54 (21)	6 months	22 (40)	11 (50)
Khawaja, 2016 **(31)**	316	<3 = 276 (87)<br>≥ 3 = 40 (13)	30 days	37 (92)	24 (65)
Koifman, 2014 (32)	537	< 2 = 475 (89)<br>≥ 2 =62 (11)	Hospital discharge	62 (100)	20 (32)
Lindman, 2015 **(22)**	507	mild = 372 (73)<br>mod = 117 (23)<br>severe = 18 (3)	30 days and<br>1 year	111 (82)<br>64 (47)	34 (31)<br>19 (30)
Little, 2017 (33)	53	severe = 53 (100)	30 days and<br>1 ano	50 (94)<br>34 (64)	34 (68)<br>21 (62)
Omar, 2020 **(23)**	174	mild = 124 (71)<br>mod = 34 (19)	Hospital discharge	59 (100)*	30 (51)
Pibarot, 2020 (34)	496	< mod= 464 (98)<br>≥ mod = 8 (2)<br>severe = 16 (9)	30 days	8 (100)	4 (50)
Schwartz, 2016 **(24)**	519	mild = 460 (89)<br>mod = 44 (8)<br>severe 15 (3)	6 months	41 (69)	24 (59)
Winter, 2020 (35)	429	non severe = 354 (82)<br>severe = 75 (18)	33 (16–48) months	75 (100)	32 (43)
Worku, 2018 **(29)**	369	mild = 311 (84)<br>mod = 28 (7)<br>severe = 30 (8)	30 days and<br>1 year	56 (96)<br>41 (70)	20 (36)<br>12 (29)
Yoshida, 2019 (36)	100	≥ mod = 100 (100)	1 year	100 (100)	47 (47)

Legend: Values are median (min-max) or n (%).

mod = moderate tricuspid regurgitation, Ref. # = reference number, TAVR = transcatheter aortic valve replacement, TR = tricuspid regurgitation.

* Post-TAVR, some patients had worsened TR severity from mild to moderate or severe TR.

SUPPLEMENTAL FIGURES

Figure 1 – "Leave one out" sensitivity analysis for comparison of all-cause mortality between none/mild and moderate/severe TR baseline degrees

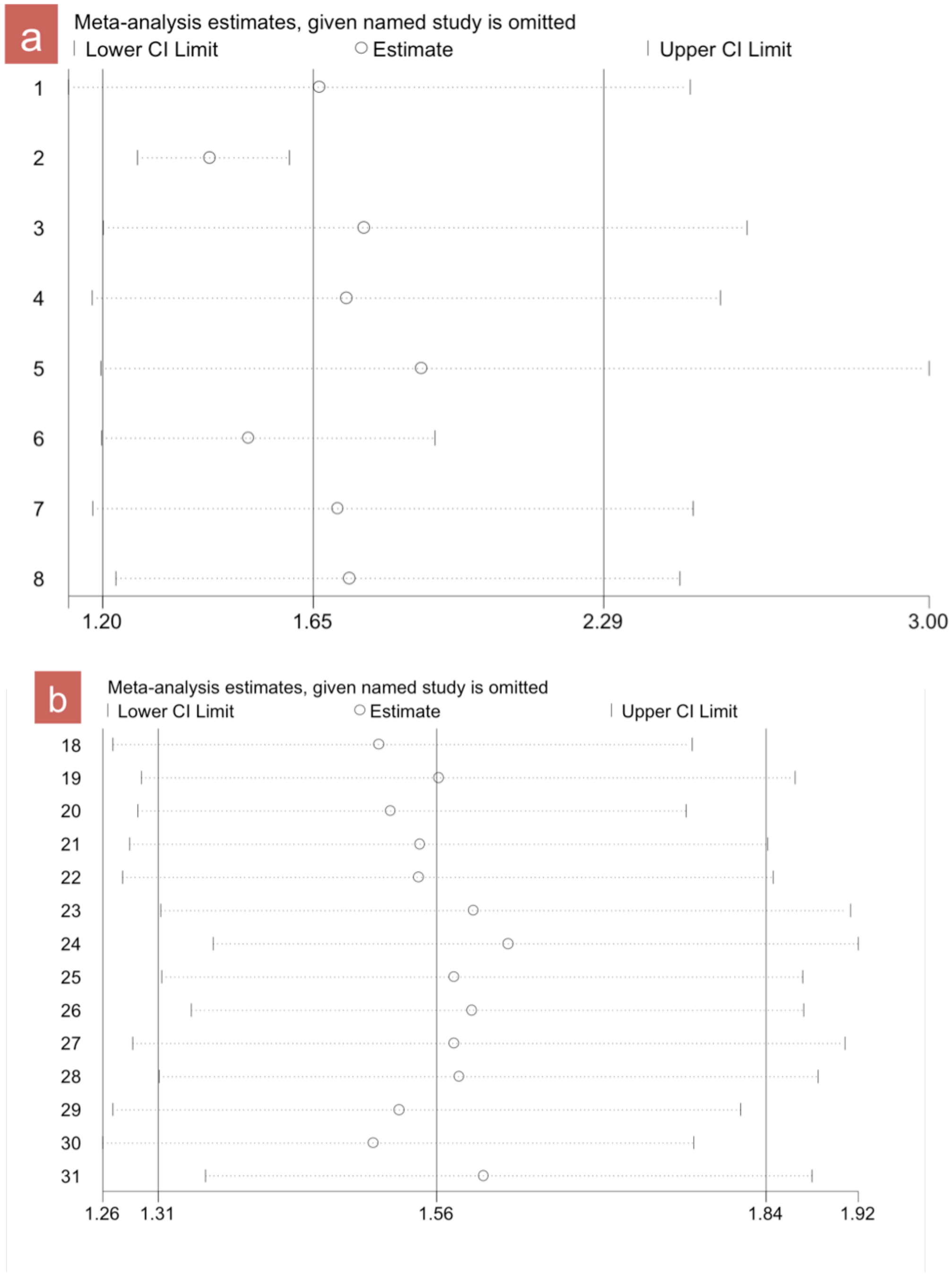

Legend: a) short-term follow-up; b) long-term follow-up

CI = confidence interval

Figure 2 – Forest plot of subgroup analysis based on reported risk estimates

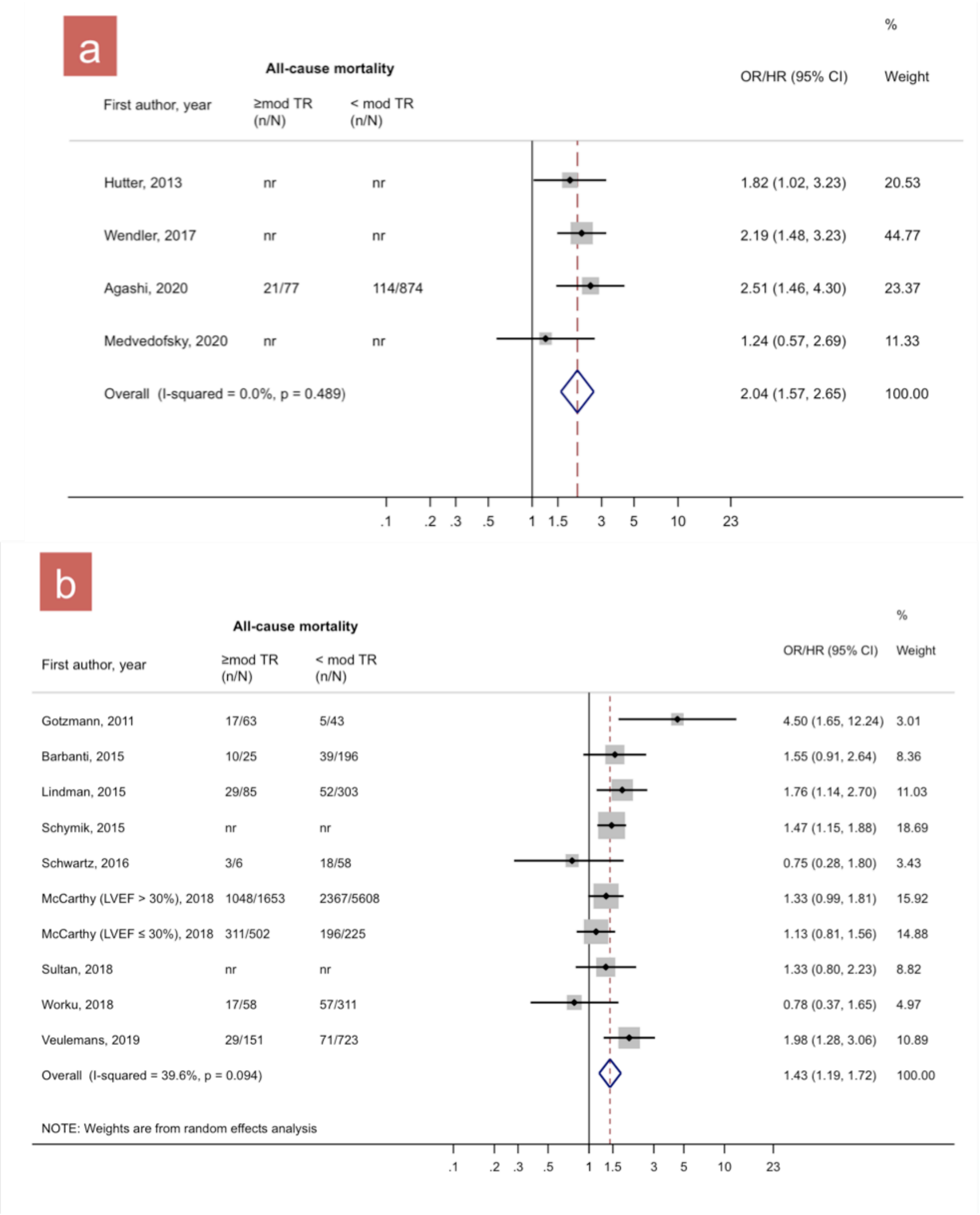

Legend: a) univariate risk estimates; b) multivariate risk estimates

CI = confidence interval, LVEF = left ventricular ejection fraction, HR = hazard ratio, mod = moderate tricuspid regurgitation, OR = odds ratio, nr = non reported, TR = tricuspid regurgitation.

Figure 3 – Funnel plots of the primary meta-analysis

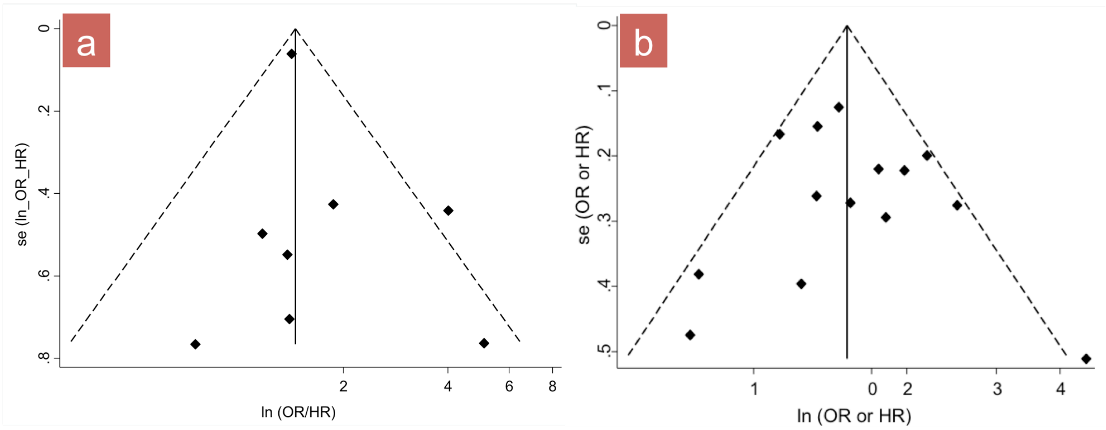

Legend: a) short-term follow-up; b) long-term follow-up

HR = hazard ratio, OR = odds ratio, SE = standard error

Figure 4 – "Leave one out" sensitivity analysis for comparison of all-cause mortality between persistence and improvement of TR grades post-TAVR

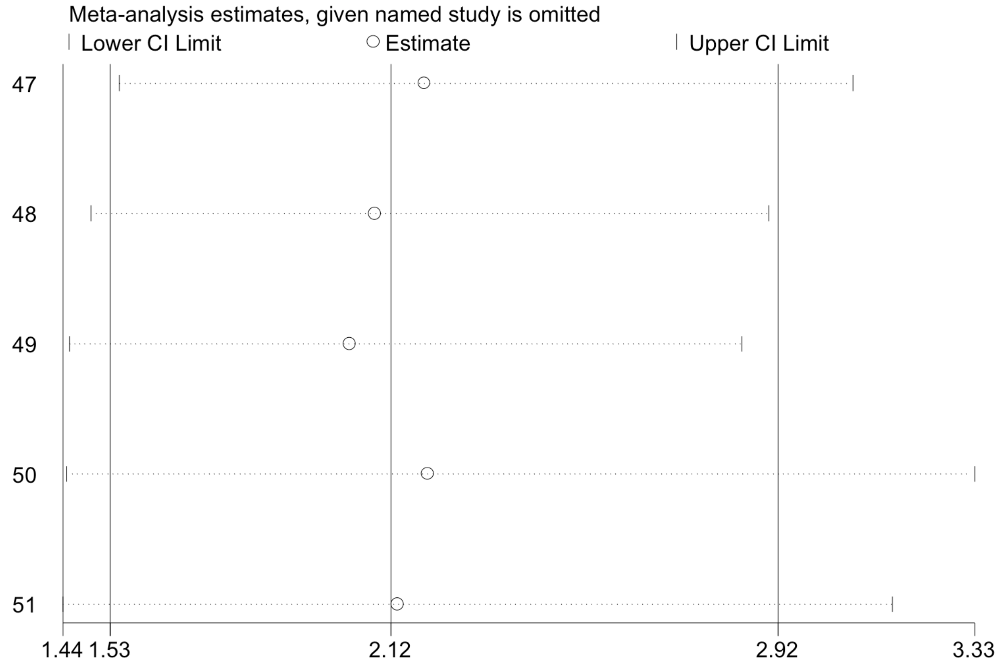

Legend: CI = confidence interval

Figure 5 – Funnel plot of the additional meta-analysis

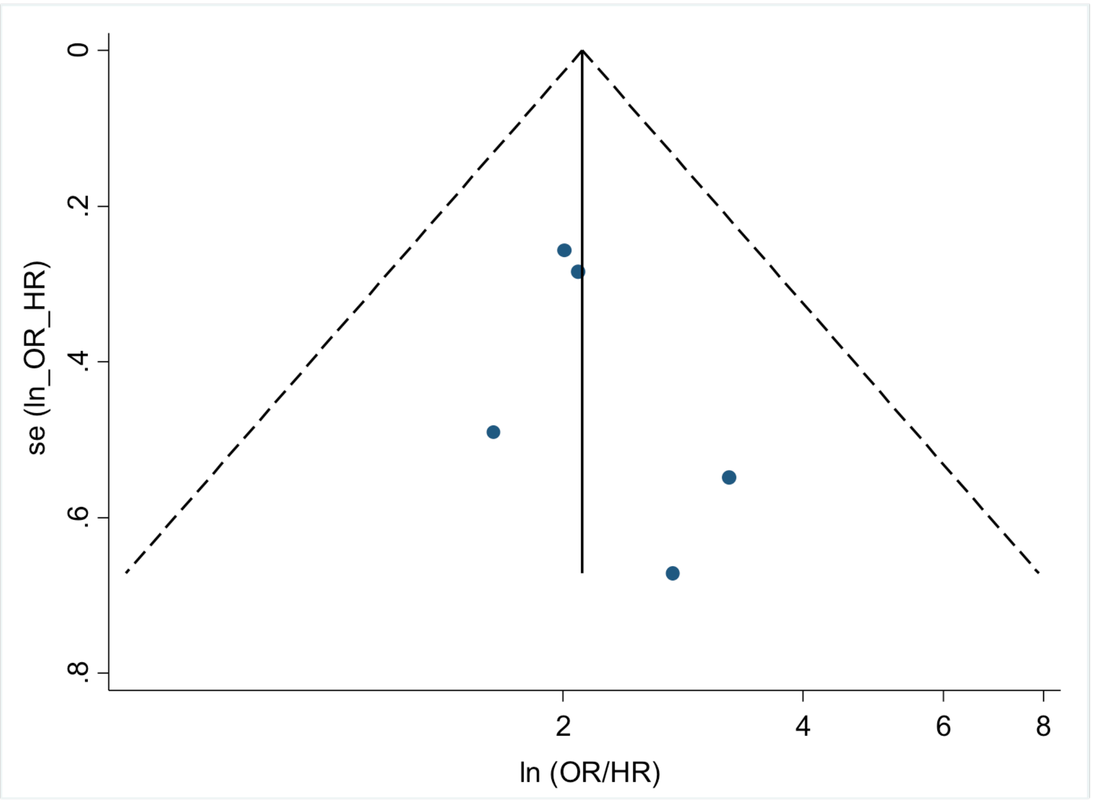

Legend: HR = hazard ratio, OR = odds ratio, SE = standard error
