# Supplementary material for: Insuficiência Tricúspide e Mortalidade em Pacientes Submetidos à Troca da Valva Aórtica Transcateter: Uma Revisão Sistemática e Metanálise
Source: Arq Bras Cardiol. 2023 Jul 27;120(7):e20220319. [Article in Portuguese] doi: 10.36660/abc.20220319 (PMC10382153; doi:10.36660/abc.20220319)
Supplement: Translation

# Translation: Tricuspid Regurgitation and Mortality in Patients Undergoing Transcatheter Aortic Valve Replacement: A Systematic Review and Meta-Analysis

**Authors:** Bruna Olandoski Erbano, Nicolle Amboni Schio, Renato Delascio Lopes, Tiago Costa Bignoto, Marcia Olandoski, Raquel Silva Brito da Luz, Guilherme Dagostin de Carvalho, Lucas Henrique Olandoski Erbano, Auristela Isabel de Oliveira Ramos, Fausto Feres, José Rocha Faria, Cristina Pellegrino Baena, Dimytri Alexandre de Alvim Siqueira

**Affiliations:** 1 Brazil Programa de Pós-Graduação do Instituto Dante Pazzanese de Cardiologia associado à Universidade de São Paulo, São Paulo, SP – Brazil; 2 Brazil Pontifícia Universidade Católica do Paraná, Curitiba, PR – Brazil; 3 USA Duke University Hospital, Durham, North Carolina – USA; 4 Brazil Instituto Dante Pazzanese de Cardiologia, São Paulo, SP – Brazil

**Keywords:** Transcatheter Aortic Valve Replacement, Tricuspid Valve Insufficiency, Mortality, Aortic Valve Stenosis

## Abstract

**Background:**

The extent of cardiac damage associated with aortic stenosis has important prognostic implications after transcatheter aortic valve replacement (TAVR). However, the role of tricuspid regurgitation (TR) in this clinical setting is still unclear.

**Objectives:**

To explore the association between TR and mortality in patients undergoing TAVR and assess changes in TR severity post TAVR and its relationship with short and mid-term mortality.

**Methods:**

Relevant databases were searched for articles published from inception until August 2020. Out of 414 screened studies, we selected 24 that reported the degree of TR pre or post TAVR. The primary outcome was all-cause mortality, and random effects meta-analysis models were conducted (at a significance level of 5%).

**Results:**

Seventeen studies reported associations between pre-TAVR TR and all-cause mortality (> 45,000 participants) and thirteen accessed TR severity post TAVR (709 participants). Moderate/severe baseline TR was associated to higher all-cause mortality both at 30 days (HR 1.65; 95% CI, 1.20-2.29) and 1.2 years (HR 1.56; 95% CI, 1.31-1.84). After TAVR, 43% of patients presented a decrease of at least one grade in TR (30 days, 95% CI, 30-56%), sustained at 12.5 months in 44% of participants (95% CI, 35-52%). Persistence of significant TR was associated with a two-fold increase in all-cause mortality (HR 2.12; 95% CI, 1.53-2.92).

**Conclusions:**

Significant TR pre TAVR is associated with higher mortality. Although TR severity may improve, the persistence of significant TR post TAVR is strongly associated with increased mortality. Our findings highlight the importance of a detailed assessment of TR pre and post TAVR and might help identify patients who may benefit from more careful surveillance in this scenario.

**Central Illustration:** : Tricuspid Regurgitation and Mortality in Patients Undergoing Transcatheter Aortic Valve Replacement: A Systematic Review and Meta-Analysis

## Introduction

In the past two decades, mortality after transcatheter aortic valve replacement (TAVR) has decreased.^1^ However, 5 years after TAVR, a mortality rate of almost 50% is attributed to cardiovascular causes.^2^ As described by Genereux et al.,^3^ the extent of cardiac damage secondary to aortic stenosis (AS) has important prognostic implications after AVR. In this regard, the association of mitral regurgitation (MR) severity and higher mortality rates after TAVR has been extensively studied.^4^ However, in a mild-to-moderate MR subgroup, tricuspid regurgitation (TR) was the prominent factor associated with a worse prognosis.^5^

Indeed, AS together with moderate/severe TR and/or pulmonary hypertension is associated with 21.3% of 1-year all-cause mortality regardless of AS treatment.^3^ However, a large registry concluded that TR was only predictive of death after TAVR in patients with more than 30% left ventricular ejection fraction (LVEF),^6^ meaning the interplay between these valvopathies remains unclear. Nonetheless, little is known about changes in TR severity over time after TAVR. The aims of this systematic review and meta-analysis were to explore the association between TR and mortality in patients undergoing TAVR, and to assess changes in TR severity post TAVR and its relationship with short- and mid-term mortality.

## Methods

### Search Strategy

This study was conducted in accordance with the Preferred Reporting Items for Systematic Reviews and Meta-Analyses (PRISMA),^7^ Meta-analyses of Observational Studies in Epidemiology (MOOSE),^8^ and Cochrane^9^ recommendations, and it was considered exempt from approval by an Institutional Review Board. Five electronic databases (MEDLINE/PubMed, SCOPUS, EMBASE, Web of Science, and LILACS) were searched for relevant articles using the following terms: TAVR OR AND tricuspid regurgitation AND prognosis/mortality ( Supplemental Material 1 ). The search was performed from inception to August 2020, with no language restrictions. Figure 1 displays the PRISMA flow diagram. Two pairs of authors independently screened all titles and abstracts, and relevant records were selected for full review. Disagreements were resolved by consensus after consulting a senior reviewer. The reference lists of the retrieved papers and relevant reviews were also screened. Kappa statistics were used to determine the degree of inter-reviewer agreement.

**Figure 1:** – PRISMA flowchart. PRISMA = Preferred Reporting Items for Systematic Reviews and Meta-Analyses. TAVR: transcatheter aortic valve replacement; TR: tricuspid regurgitation.

### Eligibility Criteria

We included studies that: (1) evaluated patients with TAVR due to AS; (2) reported TR grades by echocardiography (pre or post TAVR); (3) reported all-cause mortality as the primary outcome and cardiovascular mortality and hospitalization for heart failure (HF) as secondary outcomes, according to TR grade. We excluded studies that: (1) exclusively included patients with a bicuspid aortic valve and AS or those who underwent valve-in-valve procedures; (2) did not evaluate TR grades as recommended by echocardiography guidelines;^10^ or (3) had unclear reporting of variables, outcomes of interest, or combined outcomes, making it impossible to analyze the data. For quantitative analysis, we excluded studies that exclusively evaluated subgroup populations that differed from participants in the review. We selected the study with the largest sample when the same patient population was reported in multiple publications. Case reports, abstracts, reviews, editorials, and conference reports were excluded.

### Data Extraction

Data were gathered by 3 authors using a pre-defined data extraction sheet ( Supplemental Material 2 ), which included study details, baseline patient demographics, clinical and echocardiographic characteristics, and outcomes of interest. Disagreements were resolved by consensus after consulting a senior author. If the baseline patient characteristics were separated by groups, wherever possible we pooled data attributable to the whole population using mean (SD).^11^

### Mortality and TR Severity

The primary endpoint was defined as the incidence of all-cause mortality according to baseline TR degrees. Secondary endpoints included cardiac mortality and HF hospitalization. Studies were divided based on short- (endpoints evaluated until discharge or 30 days after TAVR) or mid-term follow-up (endpoints evaluated more than 30 days after TAVR).

TR grades assessed by echocardiography were classified as none/trace, mild, moderate, or severe. Our primary analyses compared moderate/severe TR with none/trace/mild TR grades. The association of incremental TR grades and survival was also examined by comparing the risk of none/trivial TR mortality to mild, moderate, and severe TR (secondary analysis). One study^12^ compared severe versus non-severe TR, which were included in the primary analysis.

For our additional analyses, improvements in TR were defined by changes of at least one grade from baseline to post-TAVR. Meta-analyses were also conducted separated by follow-up times, and all-cause mortality was compared between patients whose TR severity improved after TAVR vs those whose TR worsened or remained unchanged.

### Quality and Risk of Bias Assessments

The Newcastle-Ottawa Scale^13^ was used to assess risk of bias. Two independent reviewers classified studies as having low (nine stars), medium (seven or eight stars), or high risk of bias (six or less stars). Any discrepancies were resolved by consensus.

### Statistical analysis

The pooled estimates of all-cause mortality and 95% CIs of the included studies were obtained by random effects meta-analyses (DerSimonian & Laird method, with heterogeneity estimates taken from the Mantel-Haenszel method), given only observational studies were included.^9^ The most comprehensively adjusted or (when unavailable) unadjusted odds ratios (OR), hazard ratios (HR), and associated 95% CIs were extracted from each study. If risk estimates were unavailable, we captured the relevant data by corresponding with authors, hand-calculating based on the available information, or calculating unadjusted HR based on published Kaplan–Meier curves.^14^ We assumed HR and OR to approximate the same measure of risk.^9^ In one case,^6^ the HR corresponding to none/mild versus moderate/severe TR was calculated based on the given HR of other comparisons (none/trace vs mild, moderate, and severe TR). Pooled estimates of mean differences in pre- and post-TAVR proportions of moderate/severe TR grades were calculated to assess changes in TR from baseline to follow-up. Between-study heterogeneity was assessed with I^2^ statistic and classified as: < 25% indicated low heterogeneity and > 75% indicated high heterogeneity. Sensitivity analyses were performed by leave-one-out analysis, separating adjusted and unadjusted risk estimates. Meta-regression analyses were employed to test important covariates for the influence of potential effect modifiers. Publication bias was evaluated by funnel plot symmetry and Egger’s^15^ test (p-value > 0.05 indicated no significant bias). All analyses were performed using Stata statistical software version 14.1 (StataCorp LP, College Station, Texas, USA).

## Results

### Study selection

Electronic searches yielded 414 nonduplicate studies; 2 additional studies were selected manually. After title and abstract assessment, 88 studies were selected for full-text evaluation (Kappa = 0.86 [95% CI, 0.79-0.92]). Finally, 24 reports were deemed eligible and were included in our systematic review: 17 evaluating the impact of baseline TR on all-cause mortality after TAVR^5 , 6 , 12 , 16 - 29^ and 13 in the additional analysis.^17 , 20 , 22 - 24 , 29 - 36^ For quantitative analyses, we excluded 1 article^5^ which evaluated a specific subgroup of patients with mild to moderate MR because it considered a divergent population with a probably higher proportion of patients with primary TR. A summary of the 17 selected studies is provided in Table 1 . Eight studies reported data on 30-day outcomes, and 14 studies reported data on mid-term follow-up (mean of 1.2 years).

**Table 1:** – Main characteristics of the included studies

First author, year	Region	Number of	Study	Inclusion	No. of participants	Valve	Transfemoral			All-cause
(Ref. No.)		centers	design	period	undergoing TAVR	type (%)	access (%)	TR severity (%)	Follow-up	mortality (%)*
Agasthi, 2020	USA	3	Retrospective	January 2012 to	954	BEV 745 (78)	726 (76)	< mod = 877 (92)	1 year	135 (14)
(16)	(Mayo Clinic Hospitals)			June 2017		SEV 209 (22)		≥ mod = 77 (8)		
Amat-santos, 2018	Spain	6	Retrospective	August 2007 to	813	BEV 194 (24)	813 (100)	< 2 = 602 (74)	6 months	84 (10)
(5)				January 2015		SEV 608 (76)		≥ 2 = 208 (26)		
Barbanti, 2015	Canada	1	Retrospective	January 2007 to	518	BEV 483 (93)	343 (66)	< mod = 439 (85)	30 days and	118 (23)
(17)				August 2012		SEV 35 (7)		≥ mod = 79 (15)	2 years	
Barvalia, 2017	USA	1	Retrospective	2012 to 2015	460	BEV 280 (61)	330 (72)	Mild = 352 (76)	30 days	25 (5)
(18)	(New Jersey)					SEV 180 (39)		Moderate = 32 (7)		
								Severe = 43 (9)		
Gotzmann, 2011	Germany	1	Prospective	June 2008 to	145	SEV 145 (100)	140 (96)	Mild = 43 (30)	6 months	23 (16)
(19)	(Bochum)			September 2010				Moderate = 46 (32)		
								Severe = 17 (12)		
Hutter, 2013	Germany	1	Prospective	June 2007 to	268	BEV 74 (28)	194 (72)	< mod = 197 (78)	30 days and	108 (40)†
(20)	(Munich)			August 2009		SEV 194 (72)		≥ mod = 54 (21)	2 years	
Kjonas, 2019	Norway	2	Prospective	February 2010 to	218	BEV 170 (78)	122 (56)	< mod = 168 (77)	30 days	19 (9)
(21)				June 2013		SEV 48 (22)		≥ mod = 45 (21)		
Lindman, 2015	USA and	57	Prospective	December 2011 to	507	BEV 507 (100)	507 (100)	Mild = 372 (73)	1 year	112 (22)
(22)	Canada			November 2013				Moderate = 117 (23)		
								Severe = 18 (3)		
McCarthy, 2018	USA	365	Retrospective	November 2011 to	34576	nr	nr	None/trace = 6772 (19)	Intrahospital	3993 (11)
(6)	(STS registry)			March 2015				Mild = 19393 (56)	and 1 year	
								Moderate = 6687 (19)		
								Severe = 1724 (5)		
Medvedofsky, 2020	USA	1	Retrospective	May 2007 to	334	nr	nr	Non severe = 329 (98)	1 year	80 (24)
(12)	(Washington)			March 2014				Severe = 5 (2)		
Omar, 2020	USA	1	Retrospective	August 2014 to	174	BEV 76 (44)	166 (95)	Mild = 124 (71)	Intrahospital	13 (7)
(23)	(Florida)			January 2017		SEV 98 (56)		Moderate = 34 (19)		
								Severe = 16 (9)		
Schwartz, 2016	Israel	1	Retrospective	March 2009 to	519	nr	nr	Mild = 460 (89)	30 days and	108 (21)
(24)				June 2014				Moderate = 44 (8)	1.5 ± 1.17 years	
								Severe = 15 (3)		
Schymik, 2015	Multicenter	99	Prospective	July 2010 to	2688	BEV 2688 (100)	1685 (62)	< mod = 2089 (85)	1 year	515 (19)
(25)	(17 countries)			November 2011				≥ mod = 343 (14)		
Sultan, 2018	USA	1	Retrospective	July 2011 to	457	BEV 369 (80)	337 (74)	< mod = 387 (85)	23 ± 14 months	103 (22)
(26)	(Pittsburgh)			January 2016		SEV 87 (20)		≥ mod = 70 (15)		
Veulemans, 2019	Germany	1	Retrospective	2009 to 2018	874	nr	737 (84)	< mod = 723 (83)	1 year	100 (11)
(27)	(Düsseldorf)							≥ mod = 151 (17)		
Wendler, 2017	Europe	80	Prospective	July 2014 to	1946	BEV 1946 (100)	1694 (87)	< mod = 1470 (89)	1 year	245 (13)
(28)				October 2015				≥ mod = 180 (11)		
Worku, 2018	USA	1	Prospective	2009 to 2014	369	BEV 359 (97)	230 (62)	Mild = 311 (84)	30 days and	74 (20)
(29)	(New York)					SEV 10 (3)		Moderate = 28 (7)	610 days (mean)	
								Severe = 30 (8)		

Values are mean ± SD or n (%). All studies considered values < 0.05 to indicate statistical significance. BEV: balloon-expandable transcatheter aortic valve; mod: moderate tricuspid regurgitation; No.: number; nr: not reported; Ref.: reference; SEV: self-expandable transcatheter aortic valve; TAVR: transcatheter aortic valve replacement; TR: tricuspid regurgitation. * All-cause mortality ratio reported at longest follow-up. † All-cause mortality at 1 year follow-up.

### Study population

More than 45 000 patients from approximately 600 health centers worldwide were included. The mean age was 81.7 ± 8.5 years, 52% of them were female, the mean Society of Thoracic Surgeons (STS) score was 8.2 ± 6.0. Approximately 22% of patients had moderate or severe TR at baseline. The clinical features and baseline echocardiographic parameters are listed in Tables 2 and 3 .

**Table 2:** – Clinical characteristics of the included patients

First author, year	No. of participants										NYHA III/IV
(Ref. No.)	undergoing TAVR	Age (years)	Female (%)	STS score†	HTN (%)	DM (%)	CAD (%)	Stroke/TIA (%)	AF (%)	Pacemaker (%)	functional class (%)
Agasthi, 2020	954	80.9 ± 8.7	392 (41)	8.2 ± 5.2	810 (85)	337 (35)	226 (27)‡	91 (9)	410 (43)	149 (16)	720 (75)
(16)											
Amat-santos, 2018	813	81 ± 7	522 (64)	6.9 ± 5.1	660 (82)	306 (38)	327 (41)	nr	201 (27)	nr	431 (72)
(5)											
Barbanti, 2015	518	81.5 ± 8.4	233 (45)	8.3 ± 5.2	402 (78)	156 (30)	173 (33)‡	76 (15)	198 (38)	86 (17)	449 (87)
(17)											
Barvalia, 2017	460	81.7 ± 8	251 (55)	7.6 ± 4.8	426 (97)	185 (40)	384 (84)	nr	nr	nr	nr
(18)											
Gotzmann, 2011	145	79.1 ± 6.4	nr	Logistic EuroSCORE†:	127 (88)	nr	nr	nr	nr	nr	138 (95)
(19)				21 ± 16.2							
Hutter, 2013	268	80.9 ± 6.5	167 (62)	6.3 ± 4.2	nr	nr	142 (53)	36 (13)	62 (23)	nr	268 (100)
(20)											
Kjonas, 2019	218	81.8 ± 7.1	98 (45)	5.6 ± 4.0	148 (68)	62 (28)	82 (38)‡	52 (24)	100 (46)	nr	187 (86)
(21)											
Lindman, 2015	507	84.6 ± 8.5	253 (50)	10.5 ± 5.5	458 (90)	178 (35)	322 (63)	nr	186 (37)	96 (19)	242 (48)§
(22)											
McCarthy, 2018	34576	81.7 ± 8.8	16844 (49)	8.3 ± 6.0	30737 (89)	12842 (37)	23873 (69)	4240 (12)	14199 (41)	5702 (16)	28129 (81)
(6)											
Medvedofsky, 2020	334	83 ± 8.0	197 (59)	9.2 ± 5	314 (94)	110 (33)	63 (19)‡	41 (13)	0 (0)	0 (0)	283 (88)
(12)											
Omar, 2020	174	83.5 [78.4–88.0]	84 (48)	7.3 [4.7–13.6]	159 (91)	59 (34)	80 (46)‡	18 (10)	75 (43)	nr	158 (91)
(23)											
Schwartz, 2016	519	85.6 ± 6	296 (57)	EuroSCORE†:	452 (87)	182 (35)	311 (60)	nr	85 (16)	54 (10)	483 (93)
(24)				20.5 ± 14							
Schymik, 2015	2688	81.4 ± 6.6	1550 (58)	7.9 ± 6.6	2175 (81)	791 (29)	1188 (44)	345 (13)	685 (26)	304 (11)	2057 (77)
(25)											
Sultan, 2018	457	84.0 [52.0–97.0]	222 (49)	7.8 (1.0–38.0)	408 (89)	177 (39)	171 (37)‡	nr	206 (45)	nr	444 (97)
(26)											
Veulemans, 2019	874	80.5 ± 6.1	469 (54)	6.8 ± 6.6	819 (94)	283 (32)	645 (74)	174 (20)	285 (33)	112 (13)	616 (70)
(27)											
Wendler, 2017	1946	81.5 ± 6.7	934 (48)	Logistic EuroSCORE†:	1591 (82)	575 (29)	1002 (51)	376 (19)	424 (23)	230 (12)	1378 (73)
(28)				13.96 [8.97, 22.78] - TF							
				17.83 [11.40, 29.25] - non TF							
Worku, 2018	369	86.4	193 (52)	9.8	325 (88)	122 (33)	74 (20)‡	82 (22)	142 (39)	67 (18)	234 (63)
(29)											

Values are mean ± SD or median (min-max) or [interquartile range] or n (%). AF: atrial fibrillation; CAD: coronary artery disease; DM: diabetes mellitus; EuroSCORE: European System for Cardiac Operative Risk Evaluation; HTN: hypertension; No.: number; nr: not reported; NYHA: New York Heart Association; Ref.: reference; STS: Society of Thoracic Surgeons; TIA: transient ischemic attack; TAVR: transcatheter aortic valve replacement; TF: transfemoral access. † STS and EuroSCORE are algorithms are based on the presence of coexisting illnesses in order to predict 30-day operative mortality. ‡ We considered as evidence of CAD, if not clearly stated, patients with previous myocardial infarction (MI). § Only included NYHA IV patients.

**Table 3:** – Echocardiographic characteristics of the included patients

First author, year		Mean	Mean		Mod/severe	
(Ref. No.)	Mean LVEF (%)	gradient (mmHg)	AVA (cm^2^)	PASP	MR (%)	RV dysfunction (%)
Agasthi, 2020	57.1 ± 13.1	43.2 ± 13.6	0.87 ± 0.33	42.3 ± 14.4†	46 (5)	nr
(16)						
Amat-santos, 2018	60 [52-70] - TR < 2	47 [39-56] - TR < 2	0.62 [0.5-0.8] - TR < 2	47.2 ± 16.8 - TR < 2	303 (40)	TAPSE:
(5)	60 [50-65] - TR ≥ 2	44 [36-59] - TR ≥ 2	0.64 [0.5-0.8] - TR ≥ 2	49.8 ± 16.6 - TR ≥ 2		21 [19-23] - TR <2
						20 [17-22] - TR ≥ 2
Barbanti, 2015	53.9 ± 13.9	42.2 ± 16.3	0.7 ± 0.4	43.7 ± 17.8	208 (40)	RVEDD:
(17)						39.9 ± 7.3
Barvalia, 2017	50.9 ± 14.9	47.6 ± 15.5	0.67 ± 0.24	nr	80 (17)	nr
(18)						
Gotzmann, 2011	55.8 ± 12.2	46.6 ± 13.7	nr	91 (63) - PASP > 25 mmHg	83 (57)	nr
(19)						
Hutter, 2013	44 (16) - LVEF < 35%	48.7 ± 16.7	0.64 ± 0.18	62 (23) - PASP > 60	60 (22)	45 (17)
(20)						
Kjonas, 2019	110 (50) - LVEF ≥50%	51.6 ± 14.8	0.63 ± 0.2	21 (10) - PASP > 60	45 (21)	TAPSE:
(21)	79 (36) - LVEF 31-49%					1.6 ± 0.5
	23 (10) - LVEF ≤ 30%					
Lindman, 2015	51.2 ± 12.6	45.5 ± 13.7	0.34 ± 0.09*	40 (32 - 52) - no/mild TR	147 (29)	162 (34)
(22)				44 (35-58) - mod TR		
				43 (30 - 52) - severe TR		
McCarthy, 2018	53.2 ± 14.1	44.2 ± 15.0	nr	46.2 ± 15.0†	10183 (29)	nr
(6)						
Medvedofsky, 2020	53 ± 14	49 ± 13	0.44 ± 0.09	45 ± 16	4 (1)	63 (19)
(12)						
Omar, 2020	57.5 [43–65]	42 ± 15	0.69 ± 0.2	46.0 ± 15.3†	nr	nr
(23)						
Schwartz, 2016	56.3 ± 9	46.9 ± 15	0.71 ± 0.18	42.5 ± 15	109 (21)	84 (16)
(24)						
Schymik, 2015	54.4 ± 12.5	47.6 ± 16.2	0.7 ± 0.2	44.9 ± 14.9	519 (20)	nr
(25)						
Sultan, 2018	53.9 ± 13.4	48.0 ± 15.4	0.63 ± 0.18	44.1 ± 16.8	55 (12)	TAPSE < 16:
(26)						139 (30
Veulemans, 2019	51.4 ± 12.5	37.0 ± 16.4	0.7 ± 0.2	510 (58) - PASP ≥ 25 mmHg	155 (18)	nr
(27)						
Wendler, 2017	100 (6) - LVEF <30%	44.1 ± 16.0	0.73 ± 0.210	nr	249 (14)	nr
(28)						
Worku, 2018	51.6	45.6	0.7	59.4	78 (21)	31 (8)
(29)						

Values are mean ± SD or median (min-max) or [interquartile range] or n (%). AVA: aortic valve area; LVEF: left ventricular ejection fraction; mod: moderate tricuspid regurgitation; MR: mitral regurgitation; nr: not reported; PASP: pulmonary artery systolic pressure; Ref.: reference; RV: right ventricle; RVEDD: right ventricular end diastolic diameter; TAPSE: tricuspid annular plane systolic excursion; TR: tricuspid regurgitation. * Aortic valve area indexed to body surface. † In the absence of PASP, RV systolic pressure was reported.

### Risk Estimates and Bias Assessments

Most studies reported standard comparisons (none/mild versus moderate/severe TR) for all-cause mortality. In studies with short follow-up, unadjusted OR analyses were the most commonly reported, whilst HR-adjusted comparisons were mostly reported by studies with mid-term follow-up. Although highly variable between studies, clinical and echocardiographic covariates (age, sex, STS/EuroSCORE, hypertension, diabetes, atrial fibrillation [AF], NYHA functional class, LVEF, MR, and pulmonary artery systolic pressure [PASP] were included in the models ( Supplemental Table 1 ). The overall risk of bias was low or moderate in all but one^5^ study (Kappa = 0.72 [95% CI, 0.54-0.89]) ( Supplemental Table 2 ).

### Primary Analysis: None/Mild TR vs Moderate/Severe TR

At 30 days after TAVR, moderate/severe TR was associated with an increased risk of all-cause mortality when compared to none/mild TR (HR 1.65; 95% CI, 1.20-2.29; I^2^= 25.7%; p = 0.224). After a mean follow-up of 1.2 years, the pooled analysis of 14 studies also revealed that higher grades of TR were associated with a worse prognosis (HR 1.56; 95% CI, 1.31-1.84; I^2^= 44.1%; p = 0.039) ( Figures 2 and 3 ).

**Figure 2:** – Forest plot comparing all-cause mortality (30 days) between patients with none/mild and moderate/severe TR baseline grades. CI: confidence interval; HR: hazard ratio; mod: moderate tricuspid regurgitation; OR: odds ratio; TR: tricuspid regurgitation.

**Figure 3:** – Forest plot comparing all-cause mortality (1.2 years) between patients with none/mild and moderate/severe TR baseline grades. CI: confidence interval; LVEF: left ventricular ejection fraction; HR: hazard ratio; mod: moderate tricuspid regurgitation: OR: odds ratio: TR: tricuspid regurgitation.

In the leave-one-out sensitivity analysis, risk ratios ranged from 1.20-3.0 (short term) and 1.26-1.92 (mid term), indicating that the pooled estimate was robust and not influenced by a single study. Subgroup analysis showed less heterogeneity (I^2^= 0%; p = 0.489 [unadjusted] and I^2^= 39.6%; p = 0.094 [adjusted]) when studies were pooled according to univariate/multivariate risk estimates. Meta-regression analysis revealed that the proportion of patients with significant TR in each study did not change the association between TR and all-cause mortality (p = 0.676). These analyses revealed the absence of publication bias, ie, symmetrical funnel plots and p > 0.05 for all Egger’s linear regression tests ( Supplemental Figures 1-3 ).

### Secondary Analysis: Mortality and TR Severity

At short term, we observed no statistically significant differences in all-cause mortality between patients with moderate TR and those with no/mild TR (HR 4.14; 95% CI, 0.73-23.45) despite a high heterogeneity (I^2^= 93.1%; p < 0.001). However, severe TR was associated with 83% increased mortality when compared to no/mild TR (HR 1.83; 95% CI, 1.47-2.28; I^2^= 0%; p = 0.380) ( Figure 4 ).

**Figure 4:** – Forest plot comparing all-cause mortality (30 days) in patients with increasing TR grades. CI: confidence interval; HR: hazard ratio; mod: moderate tricuspid regurgitation; OR: odds ratio; TR: tricuspid regurgitation.

At mid term (mean 318 days), when comparing patients with no/trace TR to those with mild TR (HR 0.88; 95% CI, 0.77-1.00) and moderate TR (HR 1.17; 95% CI, 0.91-1.51), no differences in mortality risk ratios were observed. However, severe TR was associated with a significantly increased risk of all-cause mortality when compared to no/trace TR (HR 1.57; 95% CI, 1.05-2.36), albeit with moderate heterogeneity (I^2^= 66.1%; p = 0.031) ( Figure 5 ).

**Figure 5:** – Forest plot comparing all-cause mortality (318 days) in patients with increasing TR grades. CI: confidence interval; LVEF: left ventricular ejection fraction; HR: hazard ratio; mod: moderate tricuspid regurgitation; OR: odds ratio; TR: tricuspid regurgitation.

### Other outcomes

Details of cardiovascular mortality and rehospitalization for HF are shown in Supplemental Table 3 . Overall, higher risk estimates of outcomes were observed in individuals with higher TR grades.

### Additional Analyses: Changes in TR Severity After TAVR

Thirteen studies^17 , 20 , 22 - 24 , 29 - 36^ comprising 709 patients reported TR pre- and post-TAVR grades. Study details and patient characteristics are shown in Supplemental Table 4 . Except for two studies^31 , 35^ (which evaluated only severe TR grades), all studies reported changes in moderate/severe TR grades post-TAVR. Eight studies reevaluated TR grades in the short term (up to 30 days) and eight studies revisited TR grades after 30 days (mean = 12.5 months).

By day 30 after TAVR, TR severity reduced by at least one grade in 43% of patients (95% CI, 0.30-0.56; I^2^= 85.6%; p < 0.001). At a mean follow-up of 12.5 months, 44% of patients showed improvements in TR grades post TAVR (95% CI, 0.35-0.52; I^2^= 61.6%; p = 0.01) ( Figures 6 and 7 ). Meta-regression analyses revealed that improvements in TR grades (at short and mid terms) were not influenced by the proportion of patients with AF or RV (right ventricular) dysfunction, or by PASP values (p > 0.05 for all).

**Figure 6:** – Changes in moderate/severe TR grades at 30 days post TAVR. CI: confidence interval; ES: effect size; TR: tricuspid regurgitation.

**Figure 7:** – Changes in moderate/severe TR grades at mid term post TAVR. CI: confidence interval; ES: effect size; TR: tricuspid regurgitation.

In the pooled analysis, the persistence of moderate/severe TR grades after a mean follow-up of 21 months post TAVR was associated with all-cause mortality (HR 2.12; 95% CI, 1.53-2.92; I^2^= 0%, p = 0.901) ( Figure 8 ). No significant changes were detected in the overall effect size after performing a leave-one-out sensitivity analysis, and no evidence of publication bias across studies was recorded ( Supplemental Figures 4 and 5 ).

**Figure 8:** – Forest plot comparing all-cause mortality between patients with persistence and improvement of TR grades post TAVR. CI: confidence interval; HR: hazard ratio; OR: odds ratio.

## Discussion

This meta-analysis of 23 studies including more than 45 000 patients and evaluating the association between TR and clinical outcomes after TAVR has three main findings. First, moderate or severe TR at baseline was associated with increased all-cause mortality, both at 30 days and at mid term (1.2 years); second, a gradient was seen between TR severity and mortality. Patients with severe TR had at least a 57% increased risk of death in the mid term (318 days) when compared to those with none/trivial TR. Third, after TAVR, TR severity improved by at least one grade in > 40% of patients. Patients without improvements in TR severity post procedure presented worse outcomes. Our results confirm the main findings of similar meta-analyses in this field.^37 - 39^ Uniquely, besides including data from a recent and large registry from the STS,^6^ we evaluated the association between change in TR degree and subsequent mortality and have demonstrated a gradient with the highest mortality seen among patients with severe TR. Finally, we also analyzed changes in TR severity after TAVR and the association between persistent significant TR and survival.

The relationship between concomitant TR and prognosis has received limited attention in mainstream TAVR studies, and controversial findings have been described. While several reports^18 , 19 , 28^ have suggested increased mortality when significant TR was detected pre-procedure, others observed that this association was no longer significant after multivariable adjustment^20 , 21 , 24 , 25 , 29^ or that it existed only when significant TR persisted following TAVR,^22 , 24 , 29 , 36^ regardless of baseline TR severity.^22^ Although patients with moderate/severe TR had more comorbidities and higher risks,^6 , 17 , 20 , 22 , 24 , 29^ after pooling the results of all multivariable adjustments, the presence of significant TR remained related to worse prognosis after TAVR in our meta-analysis.

Whether TR represents a surrogate marker of late disease or a risk factor itself remains unclear. The independent relationship between TR and worse prognosis after TAVR was reported in scenarios of LVEF greater than 30-40%^6 , 17^ or lower MR grades,^5 , 22^ possibly pointing to organic TR mechanisms not amenable to AVR.^29^ Controversially, a subgroup analysis by Gotzmann et al.^19^ suggested that the underlying etiology of TR (organic or functional) had no incremental impact on all-cause mortality post TAVR. Although we cannot assure it, since 44% of patients with TR improved by at least one grade post TAVR, it is reasonable to assume that a significant number of TR etiologies in our meta-analysis were secondary. Moreover, we observed more than twice the risk of all-cause mortality in patients with persistent moderate/severe TR after TAVR.

Sustained pulmonary hypertension,^24 , 29^ AF,^24 , 29^ tricuspid annulus diameter,^24^ and RV dilation^29^ are the main factors associated with a lack of improvement in TR after TAVR. The relationship with RV dysfunction is controversial,^24 , 29^ since perhaps RV dilation better reflects the chronicity and severity of RV overload rather than ventricular function.^22^ More important than the isolated quantification of parameters may be the combined evaluation of right ventricular-pulmonary arterial coupling, integrating the performance of the right-side unit.^26 , 40^ In our meta-regression analysis, improvements in TR grades were not influenced by the proportion of patients with AF, RV dysfunction, or by PASP values, however several criteria and methods were used to define these variables.

Recognizing the association of TR and worse prognosis post TAVR aids in clinical management and influences heart team decisions. Proper patient selection is crucial to procedure success^41 - 43^ and to date, other than reduced LVEF, there are no recommendations regarding the importance of anatomical or functional cardiac consequences of AS as a component of the AVR decision algorithm.^3^ Our findings reinforce the requirement for a careful assessment of TR before TAVR, including better risk stratifications that can identify patient subgroups where post-TAVR clinical courses are expected to be worse. These recommendations avoid TAVR-related futility, which may influence both quality of life and health care costs.^5^

As the use of TAVR is rapidly expanding, assessments of the anticipated benefits of surgical treatments for multivalvular diseases are mandatory.^6 , 22 , 24^ For surgical candidates, the addition of a tricuspid repair to open-heart surgical AVR (SAVR) may lead to better outcomes than TAVR accompanied by no TR treatment, or later isolated TR surgical repair.^5 , 6 , 22^ It is worth noting that, while current guidelines provide class I recommendations for tricuspid valve annuloplasty in this scenario,^41 - 43^ outcomes from tricuspid surgery may not be ideal.^44^ Additionally, for TR secondary to RV dysfunction, tricuspid valve repair may potentially precipitate severe RV failure secondary to increased RV afterload, in which case TAVR may be preferable to SAVR.^29^ For all reasons mentioned, proper risk stratification, careful evaluation of the tricuspid valve, and associated factors that may predict TR persistence support the TAVR versus SAVR decision and could lead to alternative transcatheter strategies of TR treatment to be tested in prospective randomized studies.

### Study limitations

Firstly, as this study was a systematic review and meta-analysis of non-randomized studies, it carried the inherent limitations of observational research, despite robust methodological rigor and sensitivity analyses. Secondly, the relatively low number of studies limited the analysis of other outcomes, rather than all-cause mortality. Thirdly, TR grade analysis was fully dependent on echocardiograms, and while most studies performed TR evaluations according to standard guidelines, others published site-reported data.^6 , 25^ Fourthly, pooling moderate and severe TR grades into one group may have combined patients with different prognoses. For this reason, we evaluated the incremental risk of each additional TR degree and showed a “dose-response” type of relationship with survival. Furthermore, it is worth mentioning that a new classification was recently proposed for TR,^45^ as it has been demonstrated that, even within patients with significant TR, mortality increases as TR becomes massive or torrential.^46^ Therefore, an analysis restratifying patients with severe TR should be addressed as well in future studies.

Despite limitations, our large study cohort and robust findings suggest the need for future randomized trials dedicated to evaluating the impact of TR on TAVR prognosis, including the investigation of factors related to TR persistence post TAVR, which as demonstrated here is associated with adverse outcomes.

## Conclusions

The presence of significant TR pre TAVR is associated with higher mortality. Although TR severity may commonly improve post TAVR, the persistence of significant TR is strongly associated with increased mortality. Our findings highlight the importance of TR pre and post TAVR and might help identify patients who may benefit from more careful surveillance in this clinical setting.

## *Supplemental Materials

For additional information, please click here.
